# *CircZBTB46* Protects Acute Myeloid Leukemia Cells from Ferroptotic Cell Death by Upregulating SCD

**DOI:** 10.3390/cancers15020459

**Published:** 2023-01-11

**Authors:** Fei Long, Zhi Lin, Qinpeng Long, Zhixing Lu, Kaiyu Zhu, Mingyi Zhao, Minghua Yang

**Affiliations:** 1 Department of Gastrointestinal Surgery, The Third Xiangya Hospital, Central South University, Changsha 410013, China; 2 Postdoctoral Research Station of Basic Medicine, The Third Xiangya Hospital, Central South University, Changsha 410013, China; 3 Department of Pediatrics, The Third Xiangya Hospital, Central South University, Changsha 410013, China; 4 Department of Pediatrics, The First Affiliated Hospital, University of South China, Hengyang 421001, China; 5 Department of Gastrointestinal, Hernia and Enterofistula Surgery, People’s Hospital of Guangxi Zhuang Autonomous Region, Nanning 530022, China; 6 School of Basic Medical Science, Central South University, Changsha 410078, China; 7 Hunan Clinical Research Center of Pediatric Cancer, Changsha 410078, China

**Keywords:** circular RNA, ZBTB46, SCD, acute myeloid leukemia, oxidative stress, ferroptosis

## Abstract

**Simple Summary:**

It is urgent to identify new biomarkers for diagnosis, prognostication, and therapeutic targets of acute myeloid leukemia (AML) so as to develop more effective surveillance and treatment programs. Ferroptosis is a crucial, iron-dependent regulated cell death driven by excessive accumulation of lipid hydroperoxides. Accumulating evidence has proven that dysregulated ferroptosis is implicated in tumor progression and is becoming an emerging therapeutic target for the treatment of AML. However, the effect of circRNAs on ferroptosis and the development of AML remain unclear. In this study, we provide the first line of evidence that *circZBTB46* is an important oncogenic circRNA as well as a diagnostic or prognostic biomarker for AML. *CircZBTB46* exerts a critical role in promoting the expression of SCD to protect AML cells from ferroptotic cell death. Importantly, our findings may offer a potential therapeutic target, *circZBTB46*, to broaden treatment options for human AML, especially concerning the use of combined treatment with ferroptosis inducers.

**Abstract:**

Circular RNAs (circRNAs) have been shown to be closely linked to the tumorigenesis and treatment response of hematological malignancies. However, the biological functions and clinical implications of circRNAs in acute myeloid leukemia (AML) remain largely unknown. CircRNA microarray datasets were analyzed to screen differentially expressed circRNAs in AML patients. It was found that *circZBTB46* was significantly upregulated in AML patients and AML cells. Moreover, the expression of *circZBTB46* was associated with the stages of AML patients and showed high sensitivity and specificity for diagnosing AML. Silencing of *circZBTB46* inhibited AML cell proliferation and induced cell cycle arrest. Importantly, the depletion of *circZBTB46* notably increased ferroptosis and enhanced RSL3-induced ferroptosis in AML cells. Mechanistically, *circZBTB46* upregulated the expression of stearoyl-CoA desaturase 1 (SCD) possibly by acting as a miRNA sponge. Finally, the *circZBTB46* knockdown repressed the tumor growth of AML in vivo. In conclusion, *circZBTB46* protects AML cells from ferroptosis and promotes the proliferation by upregulating SCD, thus suggesting that *circZBTB46* may be a potential therapeutic target for AML.

## 1. Background

Acute myeloid leukemia (AML) is a clonal hematopoietic stem cell malignancy characterized by an accumulation of immature progenitor cells with arrested differentiation, thus resulting in suppression of hematopoiesis [1]. As the most common acute leukemia in adults with high mortality rates, AML is highly heterogeneous with respect to genetic and epigenetic signatures, morphology, immunophenotype, and responses to treatment [2,3]. Although advances over the past 10–15 years have dramatically improved our understanding of the molecular and phenotypic diversity of AML, the pathophysiology of AML has not yet been fully elucidated [4]. Current treatment approaches for AML patients include intensive chemotherapy, use of hypomethylating agents (HMAs), and hematopoietic stem cell transplantation (HSCT), which are generally selected according to clinical, hematological, and genetic prognostic indicators [5]. Nonetheless, most patients will ultimately fail with these standard-of-care treatments owing to resistance to treatment, which often manifests as relapse and death after remission [6]. Therefore, it is urgent to identify new therapeutic targets of AML so as to develop more effective treatment programs [7].

Circular RNAs (circRNAs) are a novel type of ubiquitous, stable, and conserved noncoding RNA (ncRNA), which, unlike linear RNAs, have covalently closed structures and lack the 3′- and 5′-ends [8]. CircRNAs often exhibit tissue- or developmental stage-specific expression patterns, and the dysregulation of circRNAs is closely linked to various cancers, including hematological malignancies [9,10]. Accumulating evidence has indicated that circRNAs bind to certain miRNAs or RNA-binding proteins (RBPs) to regulate gene transcription and translation, thus playing critical roles in various biological processes, including tumorigenesis, growth, metastasis, drug resistance, tumor metabolism, autophagy, pyroptosis, and ferroptosis [11]. Thus, revealing the novel functions and molecular mechanisms of circRNAs and exploring the potential clinical applications of circRNAs in AML will open up new prospects for AML diagnosis, prognosis, and treatment [12].

In this study, we sought to explore the roles played by circRNAs in AML. Our screening strategy recognized that hsa_circ_0002805, a circRNA derived from the zinc finger and BTB domain containing 46 (*ZBTB46*) gene (termed *circZBTB46* hereafter), was significantly upregulated in AML and was associated with stages of AML. Moreover, *circZBTB46* exhibited high sensitivity, specificity, and diagnostic simplicity by evaluation not only in bone marrow (BM) but also in peripheral blood (PB) samples. Importantly, *circZBTB46* knockdown significantly impeded the proliferation of AML cells and induced ferroptosis. Furthermore, we found that *circZBTB46* executed its regulatory functions by upregulating SCD. Therefore, inhibiting *circZBTB46* may provide a therapeutic opportunity and a unique synergistic antitumor effect with ferroptosis inducers for AML.

## 2. Materials and Methods

### 2.1. CircRNA Microarray Analysis

CircRNA microarray datasets GSE94591 (4 healthy controls and 6 AML patients) and GSE163386 (4 healthy controls and 5 AML patients) were downloaded from the Gene Expression Omnibus (GEO) database. Both of the microarray datasets used the Arraystar human circRNA microarray to analyze the circRNAs’ profiles in bone marrow samples as based on the GPL19978 platform. Differentially expressed circRNAs (*p* < 0.05) between AML patients and healthy subjects were identified through bioinformatic analysis. A cutoff fold change (FC) of 4 was applied to select the markedly upregulated circRNAs in AML.

### 2.2. Human Samples

Peripheral blood (PB) samples were collected from 25 AML patients and 25 age- and gender-matched (1:1) healthy subjects at the Third Xiangya Hospital of Central South University. Bone marrow (BM) samples were obtained from 18 out of the 25 AML patients and 9 out of the 25 healthy volunteers. After standard treatment, 12 out of the 25 AML patients achieved complete remission (CR), and 7 out of the 12 CR patients relapsed from remission during the follow-up, whose PB samples were also collected. All clinical samples were obtained with the informed consent of the patients, and the study was approved by the Ethics Committee of the Third Xiangya Hospital of Central South University (No. 22186) according to the principles of the Declaration of Helsinki.

### 2.3. Cell Lines and Cell Culture

Human AML cell lines (HL-60, K562, and THP-1), and the human BM stromal cell line (HS-5) were purchased from the American Type Culture Collection (ATCC; Manassas, VA, USA) and were cultured in RPMI 1640 (HyClone, Logan, UT, USA) with 10% fetal bovine serum (FBS; Gibco, Waltham, MA, USA). All cells were cultured at 37 °C in a humidified atmosphere containing 5% CO_2_.

### 2.4. RNA Extraction, and Quantitative Reverse Transcription–PCR (qRT–PCR)

Total RNA was extracted using TRIzol^®^ Reagent (Invitrogen, Waltham, MA, USA) according to the manufacturer’s instructions. Nuclear and cytoplasmic RNA were isolated from AML cells using a Cytoplasmic & Nuclear RNA Purification Kit (Norgen Biotek, Thorold, ON, Canada). Reverse transcription was performed using ReverTra Ace qPCR RT Master Mix with gDNA Remover (TOYOBO, Osaka, Japan), and qRT–PCR was performed using a KOD SYBR^®^ qPCR Mix Kit (TOYOBO, Japan) according to the manufacturer’s protocol. The relative expressions of circRNAs and mRNAs were calculated using the 2^−ΔCt^ method (ΔCt = Ct*_circZBTB46_* − Ct*_GAPDH_*), whereas the fold change (FC) was calculated using the 2^−ΔΔCt^ method (ΔΔCt = ΔCt_test_ − ΔCt_control_), and *GAPDH* was utilized as an internal control. All primer pairs were designed and synthesized by Tsingke Biotechnology Co., Ltd. (Beijing, China), and the sequences of the primers are listed in Table 1.

### 2.5. RNase R and Actinomycin D Treatment Assays

For the RNase treatment assay, 2 μg of total RNA was incubated with 3 U/μg RNase R (Epicenter Technologies, Madison, WI, USA) at 37 °C for 30 min, and RNase-free water was used as a control (Mock). The digested RNA was subsequently purified with an RNeasy MinElute Cleaning Kit (Qiagen, Germantown, MD, USA), and then qRT–PCR was performed to detect the abundance of *circZBTB46* and *ZBTB46* mRNA. For the actinomycin D assay, AML cells were exposed to 3 µg/mL actinomycin D (Sigma-Aldrich, St. Louis, MO, USA) to block gene transcription for 4, 8, 12, and 24 h. Then, the cells were harvested, the total RNA was extracted, and the half-lives of the *circZBTB46* and *ZBTB46* mRNA were analyzed using qRT–PCR.

### 2.6. Small Interfering RNAs (siRNAs), Short Hairpin RNAs (shRNAs), Lentiviral Vector Construction, and Cell Transfection

Two siRNAs targeting the head-to-head splicing junction of *circZBTB46* were designed and synthesized by GenePharma (Suzhou, China). Lentivirus containing shRNA targeting *circZBTB46* were purchased from GeneChem (Shanghai, China). The full-length sequence of *circZBTB46* was amplified and cloned into a circRNA-specific overexpression lentiviral vector, GV689 (GeneChem, China). SCD overexpression plasmids (GV367) were also purchased from GeneChem (Shanghai, China). Lipofectamine 3000 (Invitrogen, USA) was used for the cell transfection according to the manufacturer’s instructions. Forty-eight hours post-transfection, the knockdown or overexpression efficiencies of the corresponding genes were evaluated using qRT–PCR. The siRNA sequences are: *si-circZBTB46#1*, GCTGTCCCAGTCTGTAGAAGA-dTdT; *si-circZBTB46#2*, CACTCGCTGTCCCAGTCTGTA-dTdT.

### 2.7. Cell Counting Kit-8 (CCK-8) Assay

The proliferation of AML cells was assessed with the CCK-8 (Dojindo Laboratories, Kumamoto, Japan). Treated cells were seeded into 96-well plates containing 100 µL of medium and cultured at 37 °C with 5% CO_2_. Then, 10 μL of the CCK-8 solution was supplemented into each well at the indicated time points (0 h, 24 h, 48 h, and 72 h), and the cells were incubated at 37 °C for 2 h. Finally, the absorbance of the cells in each well was measured using a BioTek microplate reader (BioTek Instruments, Winooski, VT, USA) at a wavelength of 450 nm.

### 2.8. Cell Cycle Analysis

Treated AML cells were fixed using 70% cold absolute ethyl alcohol and stained with propidium iodide (PI; BestBio, Shanghai, China) containing RNase A for 30 min at 37 °C. Cells were analyzed for DNA content using flow cytometry (BD Bioscience, San Jose, CA, USA), and ModFit software was used for data analysis.

### 2.9. Cell Death Assay 

Cells were seeded into 6-well plates and cultured at 37 °C with 5% CO_2_. The next day, cells were incubated with the indicated treatments. After that, the cells were stained with propidium iodide (PI) for 30 min at 37 °C. Cells were also stained with DAPI to visualize the nuclei. A fluorescence microscope (Olympus, Tokyo, Japan) was used to capture images at 100× magnification.

### 2.10. Lipid Reactive Oxygen Species (ROS) and Malondialdehyde (MDA) Detection

A BODIPY 581/591 C11 probe (Thermo Fisher Scientific, Waltham, MA, USA) was used to detect lipid ROS according to the manufacturer’s protocol. Treated cells were incubated with BODIPY 581/591 C11 at a final concentration of 3 µM at 37 °C for 30 min. Then, cells were washed three times with PBS and were measured using flow cytometry (BD Bioscience, USA). The MDA levels of the treated cells were detected using an MDA colorimetric assay kit (Elabscience, Wuhan, China) according to the manufacturer’s instructions.

### 2.11. RNA-Binding Protein Immunoprecipitation (RIP)

The EZ-Magna RNA-Binding Protein Immunoprecipitation Kit (Merck, KGaA, Darmstadt, Germany) was utilized to validate the interaction between RNA and RNA-binding protein (RBP) according to the manufacturer’s instructions. In brief, cells were lysed in RIP lysis buffer, and the cell lysates were then incubated with RIP buffer containing magnetic beads conjugated with antibodies at 4 °C overnight. Finally, RNA was extracted from the RNA-protein-bead complexes and subjected to qRT–PCR to calculate enrichment. The following antibodies were used in this assay: anti-AGO2 (Proteintech, Cat No. 67934-1-Ig) and IgG (Proteintech, Cat No. 30000-0-AP).

### 2.12. RNA Pulldown Assay

The RNA pulldown assay was performed using a Pierce™ Magnetic RNA-Protein Pull-Down Kit (Pierce Biotechnology, Waltham, MA, USA) according to the manufacturer’s protocol. In brief, a total of 1 × 10^7^ AML cells were harvested and lysed. The biotin-labeled *circZBTB46*-specific probe, or NC probe, was incubated with streptavidin-coupled dynabeads at room temperature for more than 30 min to generate probe-bound dynabeads. After the treated beads were washed, the RNA complexes bound to the beads were isolated and further analyzed using qRT–PCR.

### 2.13. Western Blot (WB) Analysis and Antibodies

A total protein extraction kit (KeyGEN BioTECH, Nanjing, China) was used to extract total protein from cells, and the protein was quantified using a BCA kit (Beyotime, Shanghai, China) and following the manufacturer’s protocol. Protein samples were then separated by sodium dodecyl sulfate polyacrylamide gel electrophoresis (SDS-PAGE) and transferred onto PVDF membranes (Millipore, Burlington, MA, USA). After blocking with 5% defatted milk at room temperature for 2 h, the membranes were washed with Tris buffered saline Tween, and then incubated with primary antibodies at 4 °C overnight. The next day, the membranes were incubated with a secondary antibody at room temperature for 1 h, followed by the visualization of the signals on the membranes using an Odyssey CLx Infrared Imaging System (LI-COR Biosciences, Lincoln, NE, USA). The following antibodies were used in this assay: anti-SCD (Proteintech, Cat No. 28678-1-AP; 1:2000 dilution) and anti-GAPDH (Proteintech, Cat No. 10494-1-AP; 1:5000 dilution). Uncropped Western blot images can be found in Figure S6.

### 2.14. Animal Experiments

Five-week-old male NOD-SCID mice were maintained under specific pathogen-free (SPF) conditions in the Department of Laboratory Animals of Central South University (Changsha, China), and procedures were performed according to the institutional ethical guidelines for animal experiments. NOD-SCID mice were randomly split into 2 groups, and the xenografted AML in these mice was generated by injecting 5 × 10^6^ *sh-NC* or *sh-circZBTB46* HL-60 cells in 150 μL of Matrigel solution into the left armpit of each mouse (*n* = 5 mice/group). Four weeks after inoculation, xenografted mice were euthanized for analysis. The tumor volume was calculated as 0.5 × length × width^2^.

### 2.15. Bioinformatics Analysis

Three open-access databases, including circBank (http://www.circbank.cn/index.html (accessed on 22 July 2022)), circInteractome (https://circinteractome.nia.nih.gov/index.html (accessed on 22 July 2022)), and ENCORI (https://starbase.sysu.edu.cn/ (accessed on 22 July 2022)), were used to predict candidate miRNAs interacting with *circZBTB46*. The target genes of miRNAs were collected from miRTarBase (https://mirtarbase.cuhk.edu.cn/ (accessed on 1 August 2022)), ENCORI (https://starbase.sysu.edu.cn/ (accessed on 1 August 2022)), and TargetScan (https://www.targetscan.org/vert_80/ (accessed on 1 August 2022)). A circRNA-miRNA-mRNA crosstalk network was built using Cytoscape software (Version 3.8.2). Gene Ontology (GO) and Kyoto Encyclopedia of Genes and Genomes (KEGG) pathway analyses for target genes were performed using the clusterProfile R package (Version 4.2.1), and the results were presented as graphs using the ggplot R package.

### 2.16. Statistical Analysis

Experiments were repeated at least three times, and one representative experiment was shown in the results. Error bars represented the mean plus or minus standard deviation of the mean. Statistical analysis was performed using either Student’s *t* test or analysis of variance (ANOVA) as indicated in the corresponding figure legends. Receiver operating characteristic curve (ROC) was used to assess the value of *circZBTB46* as a diagnostic indicator in AML. In addition, the areas under the curves (AUC) were calculated. All calculations were performed using GraphPad Prism version 8.0.1 (GraphPad Software, San Diego, CA, USA), and a two-tailed *p* < 0.05 was considered to indicate a statistically significant difference.

## 3. Results

### 3.1. CircZBTB46 Is Upregulated in AML and Associated with the Stages of AML

**Figure 1:**
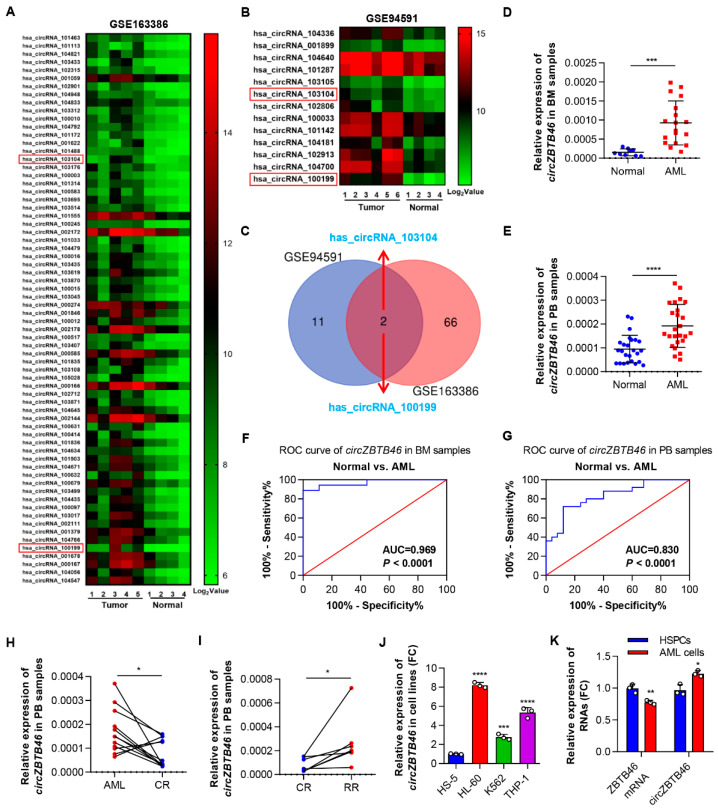
*CircZBTB46* is upregulated in AML and associated with stages of AML. (**A**) Heatmap showing the 68 most upregulated circRNAs (*p* < 0.05, Log_2_ FC > 2.0) in GSE163386 microarray datasets. (**B**) Heatmap showing the 13 most upregulated circRNAs in GSE94591 microarray datasets. High values are shown in red, whereas low values are shown in green. Each column indicates one sample, and each row indicates one circRNA. (**C**) Venn diagram showing the intersection between the most upregulated circRNAs in the two microarray datasets. (**D**) qRT–PCR showing the expression of *circZBTB46* in bone marrow (BM) samples obtained from nine healthy subjects and 18 AML patients. (**E**) qRT–PCR showing the expression of *circZBTB46* in peripheral blood (PB) samples obtained from 25 healthy subjects and 25 AML patients. (**F**) ROC curve analysis showing the diagnostic efficiency of *circZBTB46* in BM samples for distinguishing individuals with AML from healthy controls. (**G**) ROC curve analysis showing the diagnostic efficiency of *circZBTB46* in PB samples for distinguishing patients with AML from healthy subjects. (**H**) qRT–PCR showing a decreased expression level of *circZBTB46* in AML samples that achieved complete remission (CR) after treatment. (**I**) qRT–PCR showing an increased expression level of *circZBTB46* in relapsed-refractory AML patients. (**J**) qRT–PCR showing the expression of *circZBTB46* in a bone marrow stromal cell line (HS-5) and a series of AML cell lines. (**K**) qRT–PCR showing the expression of *ZBTB46* mRNA and *circZBTB46* in hematopoietic stem and progenitor cells (HSPCs) and AML cell lines. The data are shown as the mean ± SD. The *p* values were determined using a two-tailed paired (**H**,**I**) or unpaired Student’s *t* test (**D**,**E**,**K**), or a one-way ANOVA (**J**); * *p* < 0.05, ** *p* < 0.01, *** *p* < 0.001, **** *p* < 0.0001. See also Figure S1.

To investigate the circRNA expression profile in AML, we analyzed the data of GSE94591 and GSE163386 microarray datasets. As a result, 68 and 13 significantly upregulated circRNAs (*p* < 0.05, Log_2_ FC > 2.0) (Table S1) screened from the two datasets were shown in the heat map (Figure 1A,B), respectively. After taking intersection, two overlapping circRNAs (*hsa_circRNA_103104* and *hsa_circRNA_100199*) were identified (Figure 1C). Intriguingly, *hsa_circRNA_100199* (circBase ID: *hsa_circ_0012152*; gene symbol: *RNF220*) had been verified to be specifically accumulated in AML and to play a pathogenic role in AML [13,14]. Therefore, we focused on *hsa_circRNA_103104*, which has not been studied. By searching the human reference genome (GRCh37/hg19), we found that *hsa_circRNA_103104* was generated from the exons 2 and 3 regions within the *ZBTB46* gene locus; thus, we termed it as *circZBTB46*. In GSE94591 and GSE163386 microarray datasets, *circZBTB46* was remarkably increased in AML patients when compared with healthy controls (Figure S1A,B).

Next, we verified the chip results via qRT–PCR and found that *circZBTB46* expression was consistently and significantly increased in the bone marrow and peripheral blood of patients with AML as compared with healthy controls (Figure 1D,E). Moreover, ROC curve analysis showed that the AUC of *circZBTB46* in BM and PB samples were 0.969 (Figure 1F) and 0.830 (Figure 1G), respectively, for distinguishing patients with AML from healthy controls, separately, thus suggesting that *circZBTB46* has considerable value as a diagnostic indicator. Furthermore, we validated *circZBTB46* expression in AML patients at different treatment stages. A decreased expression level of *circZBTB46* was observed in AML patients who achieved complete remission (CR) after treatment (Figure 1H), whereas *circZBTB46* expression increased again in relapsed-refractory patients (Figure 1I). These results revealed the dynamic expression of *circZBTB46* following the progressive stage of AML, suggesting that *circZBTB46* might play a vital role in leukemogenesis, treatment response, and relapse. Of note, *circZBTB46* was also markedly upregulated in a series of cultured AML cell lines (HL-60, K562, and THP-1) compared with a bone marrow stromal cell line (HS-5) (Figure 1J). Additionally, *circZBTB46* expression was relatively higher in AML cells compared to hematopoietic stem and progenitor cells (HSPCs), whereas *ZBTB46* mRNA was expressed at a relatively lower level in AML cells (Figure 1K). In summary, *circZBTB46* was upregulated in AML and associated with the progression of AML.

### 3.2. Identification and Validation of circZBTB46 in AML

First of all, we verified the existence of *circZBTB46* in the circBase database (circBase ID: *hsa_circ_0002805*) and circBank database (circBank ID: *hsa_circZBTB46_002*), and we found that *circZBTB46* was an exonic circRNA generated from chr20: 62407030–62422143 with a splice length of 1255 nucleotides (nt). According to the UCSC Genome Browser (http://genome.ucsc.edu/ (accessed on 7 August 2022)), four other circRNA isoforms, namely *hsa_circ_0061176*, *hsa_circ_0061177*, *hsa_circ_0061178*, and *hsa_circ_0061179*, were identified in the *ZBTB46* gene locus (Figure S2A). Although the expression abundance of *circZBTB46* was lower than that of *ZBTB46* mRNA, *circZBTB46* was the predominant circRNA isoform in AML cell lines, which is shown in our analysis (Figure S2B,C).

**Figure 2:**
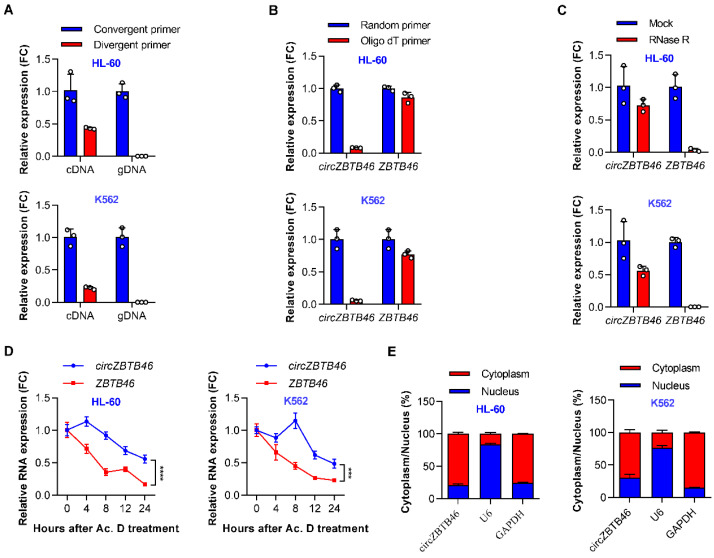
Identification and validation of *circZBTB46* in AML. (**A**) qRT–PCR showing the amplification of *circZBTB46* from cDNA or gDNA of AML cell lines using divergent primers and convergent primers. cDNA, complementary DNA; gDNA, genomic DNA. (**B**) RT–PCR using random primers or oligo dT primers showing the circular characteristics of *circZBTB46*. *ZBTB46* was used as a control for a linear RNA transcript. (**C**) qRT–PCR showing the expression of *circZBTB46* and *ZBTB46* mRNA after treatment with RNase R in AML cell lines. (**D**) qRT–PCR showing the expression of *circZBTB46* and *ZBTB46* mRNA after treatment with actinomycin D in AML cell lines. (**E**) Nuclear and cytoplasmic RNA fractionation assay showing the distribution of *circZBTB46* in AML cell lines. *GAPDH* and *U6* were utilized as positive controls in the cytoplasm and nucleus, separately. The data are shown as the mean ± SD. The *p* values were determined using a two-way ANOVA (**D**);*** *p* < 0.001, **** *p* < 0.0001. See also Figure S2.

Then, we verified the circularization of *circZBTB46* according to previously described methodology [15]. Divergent primers amplifying the circular transcripts as well as convergent primers amplifying the linear form were designed (Figure S2D,E). The qRT–PCR results showed that the circular transcripts were amplified from cDNA but not gDNA using the divergent primers (Figure 2A), indicating that *circZBTB46* was generated through the backsplicing of pre-mRNA after the transcription of gDNA. Moreover, *circZBTB46* was barely detected, but *ZBTB46* mRNA detection was unchanged when the random hexamer primers were replaced with oligo(dT)18 primers (Figure 2B), suggesting that *circZBTB46* does not contain a poly(A) tail.

Next, we investigated the stability of *circZBTB46* in AML cell lines using RNase R or actinomycin D (an inhibitor of DNA transcription) treatment assay. Resistance to digestion with RNase R exonuclease confirmed that *circZBTB46* harbored a circular RNA structure (Figure 2C). The actinomycin D treatment assay showed that the half-life of *circZBTB46* was about four times longer than that of the associated linear transcript (Figure 2D), indicating that *circZBTB46* is more stable than *ZBTB46* mRNA. Additionally, cytoplasmic and nuclear fractionation experiments showed that *circZBTB46* was mainly localized in the cytoplasm (Figure 2E). Collectively, these results confirmed that *circZBTB46* was a stable circRNA abundantly expressed in AML cells.

### 3.3. CircZBTB46 Promotes Cell Proliferation and Cell Cycle Progression of AML

**Figure 3:**
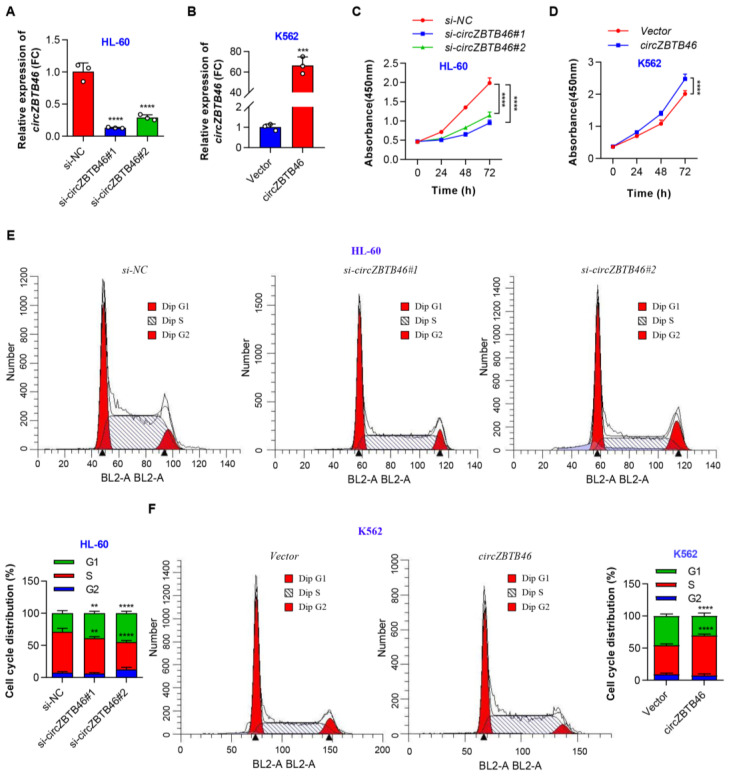
*CircZBTB46* promotes cell proliferation and cell cycle progression of AML. (**A**) qRT–PCR showing the significant knockdown of *circZBTB46* in HL-60 cells via the transfection of siRNAs. (**B**) qRT–PCR showing the significant overexpression of *circZBTB46* in K562 cells by the transfection of the circRNA-specific lentiviral plasmid. (**C**) CCK-8 assay showing the proliferation ability of HL-60 cells under control conditions (*si-NC*) or upon *circZBTB46* knockdown (*si-circZBTB46*). (**D**) CCK-8 assay showing the proliferation ability of K562 cells after the transfection of the control vector or *circZBTB46* overexpression vector. (**E**) The percentages of transfected HL-60 cells at different cell cycle phases (G1, S, and G2) were analyzed using flow cytometry. (**F**) The percentages of transfected K562 cells at different cell cycle phases (G1, S, and G2) were analyzed using flow cytometry. Data were presented as mean ± SD. Two-tailed *p* values were determined with either an unpaired Student’s *t* test (**B**,**F**) or a one-way (**A**,**E**) or two-way ANOVA (**C**,**D**); ** *p* < 0.01, *** *p* < 0.001, **** *p* < 0.0001. See also Figure S3.

To explore the biological functions of *circZBTB46* in AML, two siRNAs targeting the backsplice junction (BSJ) region of *circZBTB46* were designed, and they specifically inhibited *circZBTB46* expression (Figure 3A) and did not change the levels of *ZBTB46* mRNA in AML cells (Figure S3A). In addition, we constructed a *circZBTB46* overexpression plasmid and confirmed that *circZBTB46* was overexpressed efficiently and accurately in K562 cells (Figure 3B and Figure S3B). These results also indicated that *ZBTB46* was unaffected by *circZBTB46*.

Subsequently, in vitro studies showed that *circZBTB46* knockdown remarkably suppressed the proliferation of HL-60 cells (Figure 3C), whereas *circZBTB46* overexpression significantly accelerated cell proliferation (Figure 3D). Furthermore, the silencing of *circZBTB46* significantly increased the proportion of G1-phase cells and decreased the proportion of S-phase cells (Figure 3E). In contrast, *circZBTB46* overexpression promoted cell cycle progression of K562 cells as shown in the cell cycle analysis (Figure 3F). These data indicate that *circZBTB46* may function as an oncogene in AML cells. Additionally, we found that silence or overexpression of *circZBTB46* had no effect on *ZBTB46* expression, indicating that the regulatory effect on AML proliferation directly resulted from *circZBTB46*.

### 3.4. CircZBTB46 Protects AML Cells against Ferroptosis

**Figure 4:**
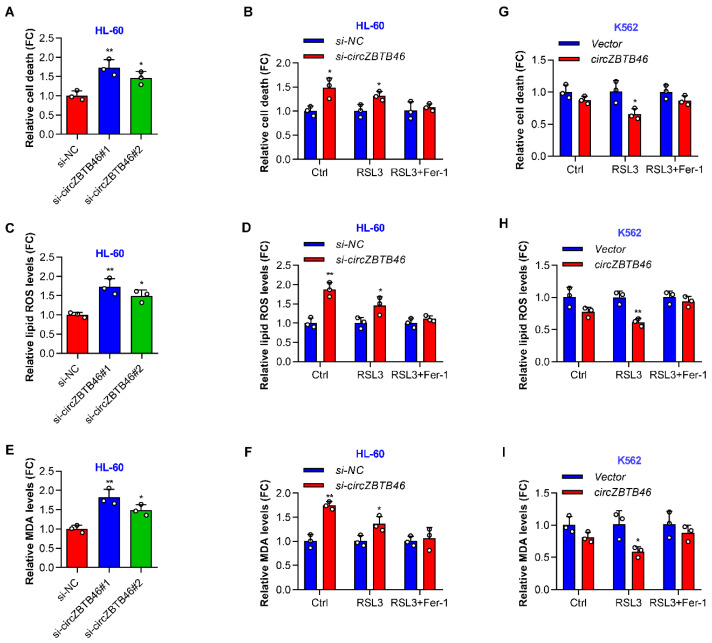
*CircZBTB46* protects AML cells against ferroptosis. (**A**) Relative cell death of HL-60 cells under control conditions (*si-NC*) or upon *circZBTB46* knockdown (*si-circZBTB46*). (**B**) Relative cell death of control and *circZBTB46*-knockdown cells following treatment with RSL3 or RSL3 + ferrostatin-1. (**C**) Relative lipid ROS levels of HL-60 cells under control conditions (*si-NC*) or upon *circZBTB46* knockdown (*si-circZBTB46*). ROS, reactive oxygen species. (**D**) Relative lipid ROS levels of control and *circZBTB46*-knockdown cells following treatment with RSL3 or RSL3 + ferrostatin-1. (**E**) Relative MDA levels of HL-60 cells under control conditions (*si-NC*) or upon *circZBTB46* knockdown (*si-circZBTB46*). MAD, malondialdehyde. (**F**) Relative MDA levels of control and *circZBTB46*-knockdown cells following treatment with RSL3 or RSL3 + ferrostatin-1. (**G**) Relative cell death of control and *circZBTB46*-overexpression cells following treatment with RSL3 or RSL3 + ferrostatin-1. (**H**) Relative lipid ROS levels of control and *circZBTB46*-overexpression cells following treatment with RSL3 or RSL3 + ferrostatin-1. (**I**) Relative MDA levels of control and *circZBTB46*-overexpression cells following treatment with RSL3 or RSL3 + ferrostatin-1. Data were presented as mean ± SD. The two-tailed *p* values were determined with either an unpaired Student’s *t* test (**B**,**D**,**F**–**I**) or a one-way ANOVA (**A**,**C**,**E**); * *p* < 0.05, ** *p* < 0.01. See also Figure S4.

As shown by cell death assay, the knockdown of *circZBTB46* induced HL-60 cell death (Figure 4A). Interestingly, the *circZBTB46*-knockdown-induced cell death was completely blocked by a ferroptosis inhibitor (ferrostatin-1) but not by the pancaspase inhibitor (ZVAD) (Figure S4A). Thus, the increased cell death of *circZBTB46*-knockdown cells was possibly mediated by ferroptosis. Moreover, the silencing of *circZBTB46* sensitized AML cells to lethality by RSL3 (a ferroptosis inducer) (Figure 4B), supporting the hypothesis that *circZBTB46* was a negative regulator of ferroptosis.

Next, we detected the lipid reactive oxygen species (ROS) to estimate the level of ferroptosis-associated lipid peroxidation in wild-type and *circZBTB46*-knockdown cells. The depletion of *circZBTB46* increased the levels of lipid ROS in HL-60 cells (Figure 4C) and exacerbated RSL3-induced lipid peroxidation (Figure 4D). Accordingly, *circZBTB46* knockdown increased the levels of malondialdehyde (MDA) in HL-60 cells (Figure 4E) and enhanced the RSL3-induced accumulation of intracellular MDA (Figure 4F), which is one of the final products of lipid peroxidation. In contrast, overexpression of *circZBTB46* decreased RSL3-induced cell death and lipid peroxidation (Figure 4G–I) despite the fact that *circZBTB46* overexpression alone had little effect on cell death, lipid ROS levels, and MDA levels in K562 cells (Figure S4B–D). Furthermore, the effects of *circZBTB46* knockdown or overexpression on RSL3-induced cell death and lipid peroxidation could be reversed by ferrostatin-1 (Figure 4B,D,F–I). Taken together, these data indicate that *circZBTB46* acted as an endogenous inhibitor of ferroptosis.

### 3.5. CircZBTB46 Exerts Its Oncogenic Effect by Upregulating SCD

**Figure 5:**
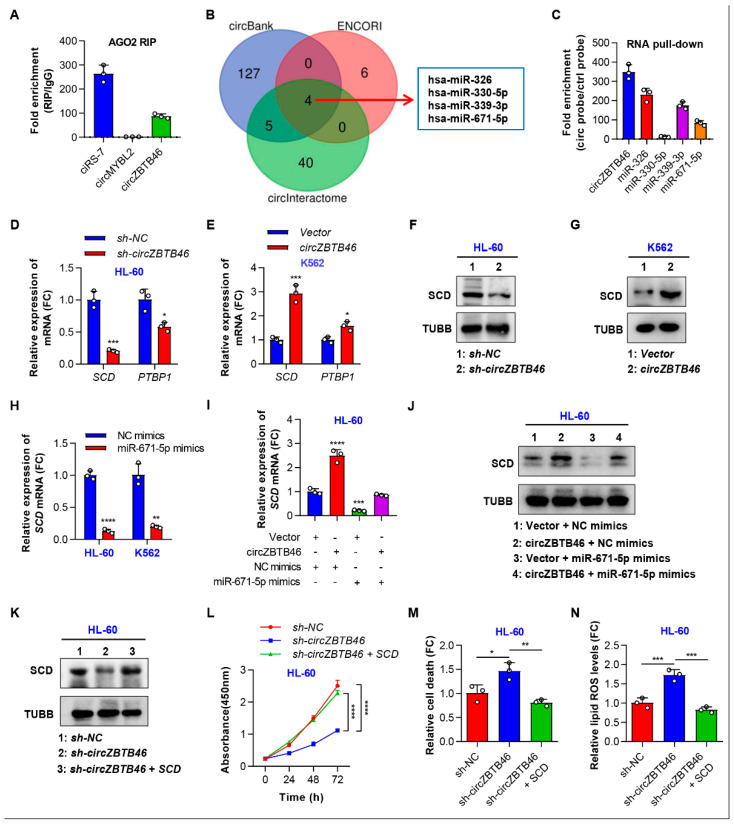
*CircZBTB46* exerts its oncogenic effect by upregulating SCD. (**A**) qRT–PCR showing the enrichment of circRNAs in a representative anti-AGO2 RIP assay in AML cells. (**B**) Venn diagram showing the intersection among the candidate miRNAs interacting with *circZBTB46* as predicted in three open-access databases. (**C**) qRT–PCR showing the enrichment of miRNAs upon *circZBTB46* pull-down in AML cell lysates. (**D**) qRT–PCR showing the expression of *SCD* and *PTBP1* mRNAs in HL-60 cells under control conditions (*sh-NC*) or upon *circZBTB46* knockdown (*sh-circZBTB46*). (**E**) qRT–PCR showing the expression of *SCD* and *PTBP1* mRNAs in K562 cells after the transfection of control vector or *circZBTB46* vector. (**F**) Representative Western blot of SCD protein in HL-60 cells under control conditions (*sh-NC*) or upon *circZBTB46* knockdown (*sh-circZBTB46*). TUBB was used as a loading control. (**G**) Representative Western blot of SCD protein in K562 cells after the transfection of control vector or *circZBTB46* vector. (**H**) qRT–PCR showing the expression of *SCD* mRNA in AML cells after the transfection of NC mimics or miR-671-5p mimics. (**I**) qRT–PCR showing the expression of *SCD* mRNA in HL-60 cells after the transfection of control vector or *circZBTB46* vector with or without NC mimics or miR-671-5p mimics. (**J**) Representative Western blot of SCD protein in HL-60 cells after the transfection of control vector or *circZBTB46* vector with or without NC mimics or miR-671-5p mimics. (**K**) Representative Western blot of SCD protein in HL-60 cells under control conditions (*sh-NC*), upon *circZBTB46* knockdown (*sh-circZBTB46*), or upon *sh-circZBTB46* + *SCD* vector cotransfection. (**L**) CCK-8 assay showing the proliferation ability of HL-60 cells under control conditions (*sh-NC*), upon *circZBTB46* knockdown (*sh-circZBTB46*), or upon *sh-circZBTB46* + *SCD* vector cotransfection. (**M**) Relative cell death of HL-60 cells under control conditions (*sh-NC*), upon *circZBTB46* knockdown (*sh-circZBTB46*), or upon *sh-circZBTB46* + *SCD* vector cotransfection. (**N**) Relative lipid ROS levels of HL-60 cells under control conditions (*sh-NC*), upon *circZBTB46* knockdown (*sh-circZBTB46*), or upon *sh-circZBTB46* + *SCD* vector cotransfection. ROS, reactive oxygen species. The data are shown as the mean ± SD. The *p* values were determined with either a two-tailed unpaired Student’s *t* test (**D**,**E**,**H**) or a one-way (**I**,**M**,**N**) or two-way ANOVA (**L**); * *p* < 0.05, ** *p* < 0.01, *** *p* < 0.001, **** *p* < 0.0001. See also Figure S5.

We then investigated the mechanism by which *circZBTB46* regulates the proliferation and ferroptosis of AML cells. Given that *ACSL4* [15], *GPX4* [16], *SLC7A11* [17], *ALDH3A2* [18], and *HMGB1* [19] are representative ferroptosis-related genes, we first examined the expression of these genes in AML cell lines with *circZBTB46* knockdown and overexpression. As shown in Figure S5A,B, silencing or overexpression of *circZBTB46* did not significantly change the expression levels of these genes in AML cell lines, indicating that *circZBTB46* may have targeted other genes to inhibit ferroptosis of AML cells. Given that cytoplasm-localized circRNAs often participate in translational regulation by acting as competing endogenous RNAs (ceRNAs), we then conducted anti-AGO2 RIP assays to determine whether *circZBTB46* functions as a miRNA sponge in AML cells. The results showed that ciRS-7 (a circRNA binding with AGO2) [20] and *circZBTB46* were significantly enriched by the anti-AGO2 antibody, but circMYBL2 (a circRNA that does not bind AGO2 [21]) was not (Figure 5A), suggesting that *circZBTB46* may interact with miRNAs. Through computational prediction, we identified four candidate miRNAs binding to *circZBTB46*, including hsa-miR-326, hsa-miR-330-5p, hsa-miR-339-3p, and hsa-miR-671-5p (Figure 5B). Further RNA pull-down assays and qRT–PCR analysis demonstrated the enrichment of hsa-miR-326, hsa-miR-339-3p, and hsa-miR-671-5p in the RNA complexes precipitated with probes against *circZBTB46* compared to those with control probes (Figure 5C). These results suggested that *circZBTB46* may act as a miRNA sponge for hsa-miR-326, hsa-miR-339-3p, and hsa-miR-671-5p in AML cells.

Next, we aimed to identify the ceRNAs of *circZBTB46*. Based on the miRTarBase, ENCORI, and TargetScan databases, a total of 581 genes were predicted as target genes of the three miRNAs (Table S2). The GO analysis showed that these target genes might play a role in biological processes such as “response to oxidative stress”, “response to nutrient levels”, “positive regulation of cell adhesion”, and so on (Figure S5C). Furthermore, the KEGG analysis showed that the target genes might participate in pathways including the “PI3K-Akt signaling pathway”, “MAPK signaling pathway”, “mTOR signaling pathway”, and so forth (Figure S5D). Additionally, the crosstalk network of circRNA-miRNA-mRNA was constructed using Cytoscape software (Figure S5E). In the ceRNA network, *circZBTB46* shared miRNA response elements (MREs) of miR-326 and miR-671-5p with HEXA, HTT, SCD, TMEM184B, MTHFR, LGALS3BP, HDLBP, and PTBP1, and shared MREs of miR-339-3p and miR-671-5p with TOMM40L, BTBD3, ANKRD52, ELK1, SH3PXD2A, CBX6, and PTBP1 (Figure S5E). Among these genes, only SCD and PTBP1 were significantly regulated by *circZBTB46* in AML cells, as shown using qRT–PCR (Figure 5D,E). WB analysis further confirmed the regulatory role of *circZBTB46* on SCD expression in AML cell lines (Figure 5F,G). Additionally, we found that miR-671-5p but not miR-326, in fact, downregulated SCD expression in AML cells (Figure 5H and Figure S5F). Importantly, the increased SCD expression induced by the overexpression of *circZBTB46* was abolished by miR-671-5p mimics (Figure 5I,J). These data indicate that *circZBTB46* might sponge hsa-miR-671-5p to upregulate SCD in AML.

To determine whether *circZBTB46* exerts its oncogenic effect by positively regulating SCD expression, we conducted in vitro rescue experiments. As shown in Figure 5K, transfection with SCD overexpression plasmids could reverse the inhibition of SCD expression mediated by *circZBTB46* knockdown. Accordingly, the decrease in cell proliferation upon *circZBTB46* knockdown was reversed after overexpressing SCD in HL-60 cells (Figure 5L). Furthermore, the significant increase in the cell death of HL-60 cells induced by *circZBTB46* knockdown was restored by transfection with SCD overexpressed plasmids (Figure 5M). Importantly, the increased lipid ROS levels resulted from *circZBTB46* knockdown were eliminated by treatment with SCD overexpression plasmids (Figure 5N). Collectively, these data indicated that *circZBTB46* may act as a ceRNA to simultaneously sponge hsa-miR-326 and hsa-miR-671-5p, thus resulting in upregulation of their downstream target, SCD, which is implicated in AML cell proliferation and ferroptosis.

### 3.6. CircZBTB46 Knockdown Impairs the Tumorigenesis of AML Cells In Vivo

**Figure 6:**
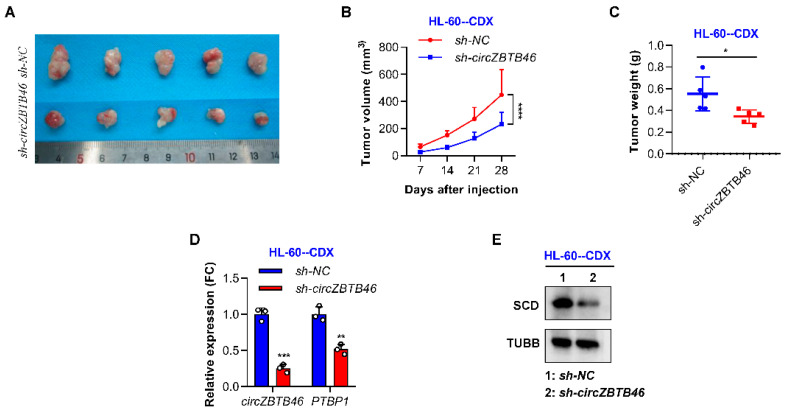
*CircZBTB46* knockdown impairs the tumorigenesis of AML cells in vivo. (**A**) Images of tumors in mice in each group (*n* = 5 mice/group). (**B**) Tumor growth curves in mice in each group. (**C**) Tumor weights in mice in each group. (**D**) qRT–PCR showing the expression of *circZBTB46* and *PTBP1* mRNA in control tumors (*sh-NC*) and *circZBTB46*-knockdown tumors (*sh-circZBTB46*). (**E**) Representative Western blot of SCD protein in control tumors (*sh-NC*) and *circZBTB46*-knockdown tumors (*sh-circZBTB46*). The data are shown as the mean ± SD. The *p* values were determined using either a two-tailed unpaired Student’s *t* test (**C**,**E**) or a two-way ANOVA (**B**); * *p* < 0.05, ** *p* < 0.01, *** *p* < 0.001, **** *p* < 0.0001.

We further addressed the effect of *circZBTB46* knockdown in vivo using NOD-SCID mice. Male NOD-SCID mice were injected subcutaneously with 5 × 10^6^ HL-60 cells with (*–circZBTB46*) or without (*sh-NC*) *circZBTB46* knockdown. The results showed that tumors derived from *circZBTB46*-knockdown cells were smaller and weighed less (Figure 6A–C), indicating that *circZBTB46* knockdown could markedly inhibit tumorigenicity of AML cells. Additionally, qRT–PCR analysis showed that the expression levels of *circZBTB46* and *SCD* mRNA were markedly decreased in tumors in the *sh-circZBTB46* group compared with those in the *sh-NC* group (Figure 6D). WB analysis further confirmed that tumor tissues with *circZBTB46* knockdown had lower expressions of SCD protein than the *sh-NC* group (Figure 6E). These results show that AML cells depended on *circZBTB46* targeting SCD and the corresponding lipid metabolism pathway for AML maintenance and progression.

## 4. Discussion 

In the present study, we first reported that *circZBTB46*, which is frequently upregulated in AML patients, is an important circRNA that regulates cell proliferation and ferroptotic cell death in AML. We revealed that *circZBTB46* protects AML cells from ferroptosis by upregulating SCD, which is a ferroptosis-protective gene. Clinically, the expressions of *circZBTB46* are associated with the treatment stages of AML patients and have potential diagnostic value in the BM or PB samples of patients with AML.

Ferroptosis, a recently described cell death process, is a crucial, iron-dependent, regulated cell death driven by excessive accumulation of lipid hydroperoxides [22]. During the process of ferroptosis, lipid homeostasis is altered with the concomitant elevation and massive accumulation of lipid-based ROS levels in cells, resulting in cell damage or even cell death [23]. Mounting evidence has proven that ferroptosis is widely involved in tumor progression and therapy resistance [24]. Ferroptosis is also important in AML. Accumulating evidence has proven that dysregulated ferroptosis is implicated in tumor progression and is becoming an emerging therapeutic target for the treatment of AML [25]. Several ferroptosis-related genes (e.g., *Aldh3a2*, *HMGB1*) have been identified and proven to play a vital role in AML [18,19]. Moreover, several promising therapeutic agents (e.g., APR-246, DHA, Neratinib, and honokiol) have been found to induce ferroptosis in AML cells [26,27,28,29]. However, the effect of circRNAs on ferroptosis and the development of AML remain unclear. As far as we know, there is only one study reporting that circKDM4C, which is downregulated in AML patients, acts as a positive regulator of ferroptosis in AML cells [30]. In the present study, we identified an upregulated circRNA in AML patients, which is associated with the tumor pathogenesis and treatment response of AML. Importantly, *circZBTB46* knockdown significantly induced ferroptosis and enhanced the lethality of ferroptosis inducer, supporting the hypothesis that *circZBTB46* acts as an endogenous inhibitor of ferroptosis in AML cells. 

The competitive endogenous RNA (ceRNA) hypothesis has attracted notable attention as a unifying function of ncRNAs as well as an alternative function of mRNAs [31]. Most cytoplasm-localized circRNAs identified to date have also been proposed to have ceRNA function, especially the highly abundant circRNAs containing many competing binding sites [32,33]. CircRNAs, such as circSPI1 [34], hsa_circ_0075451 [35], circCRKL [36], and hsa_circ_0004277 [37], may also act as miRNA sponges in AML. Of note, a circRNA may have different functions by acting as a sponge to various miRNAs rather than by containing multiple sites for just one miRNA. For instance, circSPI1 facilitated the proliferation of AML cells by simultaneously sequestering miR-1307-3p, miR-382-5p, and miR-767-5p [34]. CircCRKL contains binding sites for several miRNAs that target antioncogenes, including miR-196a-5p and miR-196b-5p, which target the p27 [36]. In our study, we found that *circZBTB46* may act as a miRNA sponge for miR-326, miR-339-3p, and miR-671-5p in AML cells, a finding based on bioinformatic analysis and validation experiments. Previous studies showed that miR-326 and miR-339-3p are significantly decreased in AML-derived samples [35,38] and that miR-326 may inhibit proliferation but promote apoptosis and phorbol myristate acetate (PMA)-induced differentiation through downregulating c-Myc in AML [39]. Thus, our study indicated that *circZBTB46* may mediate the upregulation of some pluripotency genes and play a regulatory role in AML by sponging these miRNAs. 

*CircZBTB46* shared miRNA binding sites of miR-671-5p with SCD; we then confirmed the regulatory role of *circZBTB46* on SCD in AML cells. Stearoyl-CoA desaturase (SCD), an enzyme that catalyzes the rate-limiting step in monounsaturated fatty acid synthesis, has recently been identified as a ferroptosis-related gene in various cancers, including bladder cancer [40,41], cervical cancer [42], ovarian cancer [43], and lung cancer [44]. Emerging studies showed that the inhibition of SCD induces lipid oxidation and ferroptotic cell death, and it significantly augments the antitumor effect of ferroptosis inducers (e.g., erastin and RSL3) [43,44]. In addition, SCD is linked to apoptosis, and the upregulation of SCD conferred partial protection against nicotinamide phosphoribosyltransferase (NAMPT) inhibitor-induced apoptosis in AML leukemic stem cells (LSCs) [45]. In this study, we revealed that SCD is also a ferroptosis-protective gene in AML and that overexpression of SCD reversed the ferroptotic cell death induced by *circZBTB46* knockdown. Importantly, we substantiated this concept in a mouse xenograft model. Similarly, Dong et al. found that *circKDM4C* induces ferroptosis by upregulating P53 via sponging hsa-let-7b-5p in AML [30]. Our work demonstrates that *circZBTB46* and SCD may play a key role in ferroptotic cell death and identifies *circZBTB46* or SCD inhibition as a therapeutic strategy for targeting ferroptosis in AML.

## 5. Conclusions

In conclusion, we first demonstrated that *circZBTB46* is a critical oncogene as well as a diagnostic and prognostic biomarker for AML. *CircZBTB46* exerts important functions in upregulating the expression of SCD to protect AML cells from ferroptotic cell death. Moreover, our findings offer a potential therapeutic target, *circZBTB46*, which may broaden the treatment options for patients with AML, especially for the application of combined treatments with ferroptosis inducers.

## Figures and Tables

**Table 1:** Primers used in this article.

Gene Names	Forward Primers (5′→3′)	Reverse Primers (5′→3′)
*circZBTB46* divergent primer	GTTCGAGTACCTGCCCAGAG	GGTGTCGCCTCTTCTACAGA
*circZBTB46* convergent primer	TTCAAGACGCTCTACTGCCA	GCGCTGAGTACATGAAGTCG
*ZBTB46*	CGGGAAGAAGTTCACGCGG	CTGCACACCTTGCACACATAC
*SCD*	AAACCTGGCTTGCTGATG	GGGGGCTAATGTTCTTGTCA

## Data Availability

All data supporting the findings of this study are available within the article, its supplementary information files, and from the corresponding author(s) upon reasonable request.

## Supplementary Materials

The following supporting information can be downloaded at: https://www.mdpi.com/article/10.3390/cancers15020459/s1, Figure S1: Relative expression of the two overlapping circRNAs in microarray datasets, Figure S2: Verification of the existence and circularization of *circZBTB46*, Figure S3: The expression of *ZBTB46* mRNA in AML cells with *circZBTB46* knockdown or overexpression, Figure S4: The effect of *circZBTB46* knockdown or overexpression on ferroptosis of AML cells, Figure S5: The expression of ferroptosis-related genes in AML cells with *circZBTB46* knockdown or overexpression, Figure S6: Uncropped Western blot images of Figure 5F,G,J,K and Figure 6E, Table S1: The 13 and 68 significantly upregulated circRNAs screened from the two microarray datasets, Table S2: The target genes of the three miRNAs as predicted in the miRTarBase, ENCORI, and TargetScan databases.
