# Supplementary material for: CircZBTB46 Protects Acute Myeloid Leukemia Cells from Ferroptotic Cell Death by Upregulating SCD
Source: Cancers (Basel). 2023 Jan 11;15(2):459. doi: 10.3390/cancers15020459 (PMC9857113; doi:10.3390/cancers15020459)
Supplement: Supplementary file 1 [file cancers-15-00459-s001.zip › cancers-2044453-supplementary.pdf]

cancers

MDPI

Supplementary Material: *CircZBTB46* Protects Acute Myeloid Leukemia cells from ferroptotic cell death by upregulating SCD

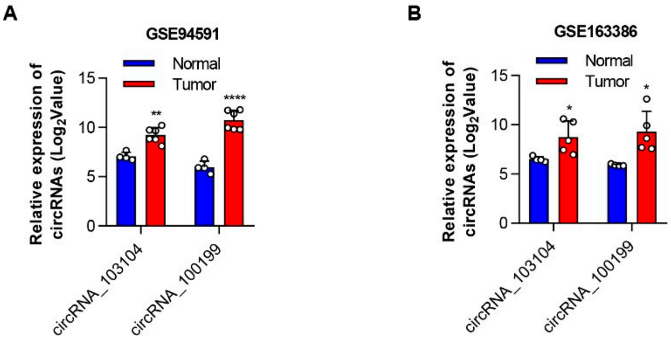

Figure S1. Relative expression of the two overlapping circRNAs in microarray datasets. (A) Relative expression of *hsa_circRNA_103104* and *hsa_circRNA_100199* in GSE94591 microarray datasets. **(B)** Relative expression of *hsa_circRNA_103104* and *hsa_circRNA_100199* in GSE163386 microarray datasets. The data are shown as the mean ± SD. The *P* values were determined by a two-tailed unpaired Student's t test; *P<0.05, **P<0.01, ****P<0.0001. Related to Figure 1.

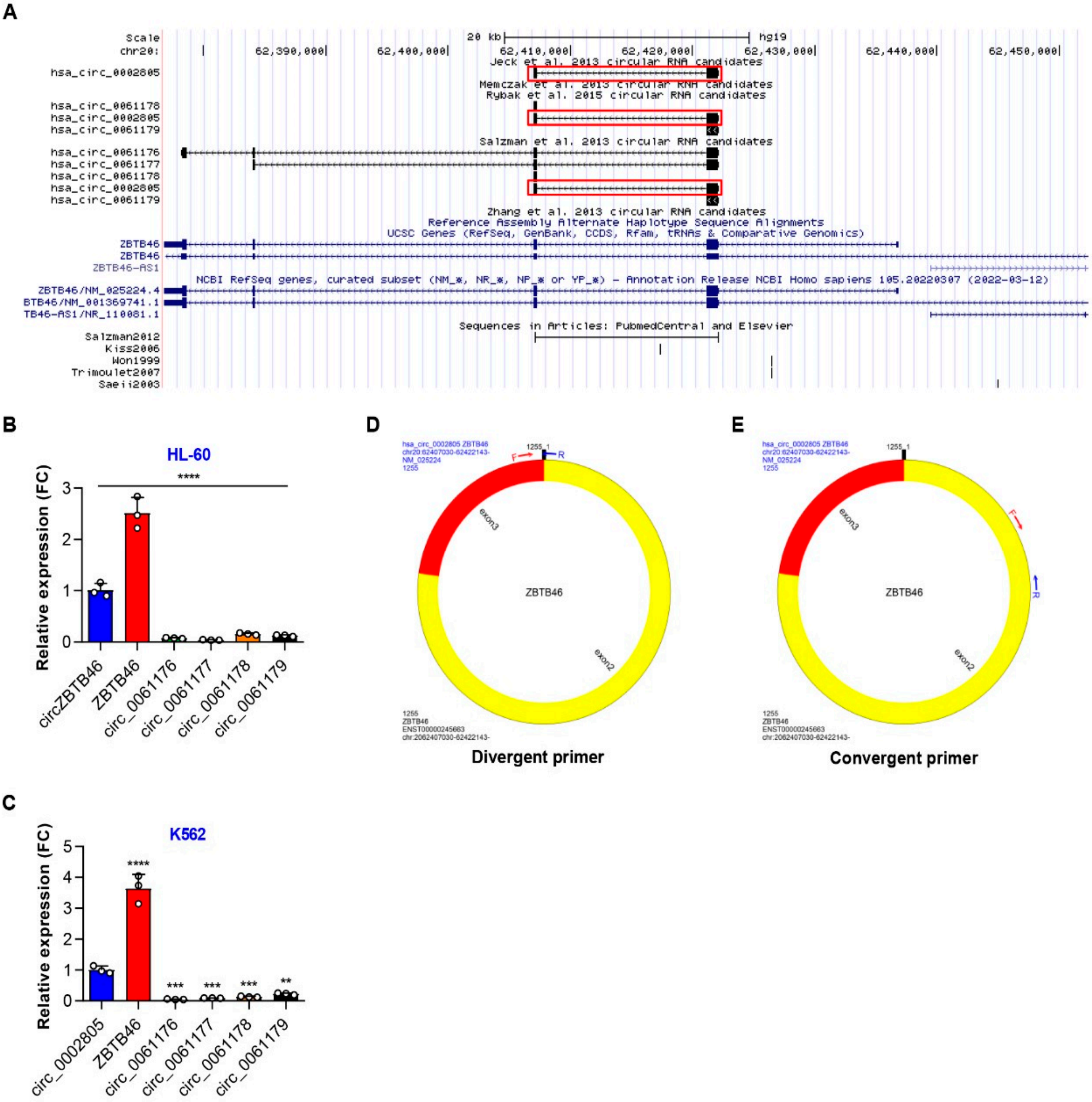

Figure S2. Verification of the existence and circularization of *circZBTB46*. (A) Schematic representation of the chromosome location of *circZBTB46* using the UCSC Genome Browser. **(B, C)** qRT–PCR showing the expression abundance of *circZBTB46*, *ZBTB46* mRNA and other homologous circRNAs in AML cell lines. **(D, E)** Schematic representation of the design of divergent and convergent primers for *circZBTB46* using circPrimer software. The data are shown as the mean ± SD. The *P* values were determined by one-way ANOVA (B and C); ***P*<0.01, ****P*<0.001, *****P*<0.0001. Related to Figure 2.

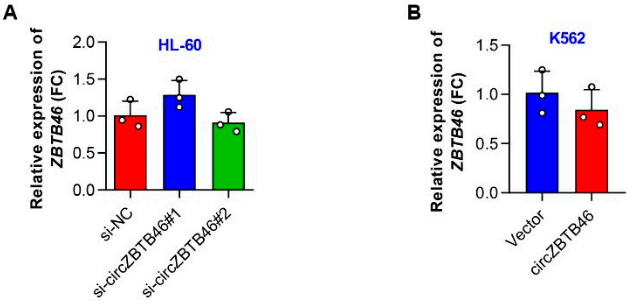

Figure S3. The expression of *ZBTB46* mRNA in AML cells with *circZBTB46* knockdown or overexpression. (A) qRT–PCR showing the expression of *ZBTB46* mRNA in HL-60 cells under control conditions (*sh-NC*) or upon *circZBTB46* knockdown (*sh-circZBTB46*). **(B)** qRT–PCR showing the expression of *ZBTB46* mRNA in K562 cells after the transfection of control vector or *circZBTB46* vector. The data are shown as the mean ± SD. The *P* values were determined by a two-tailed unpaired Student's t test (B), or one-way ANOVA (A); Related to Figure 3.

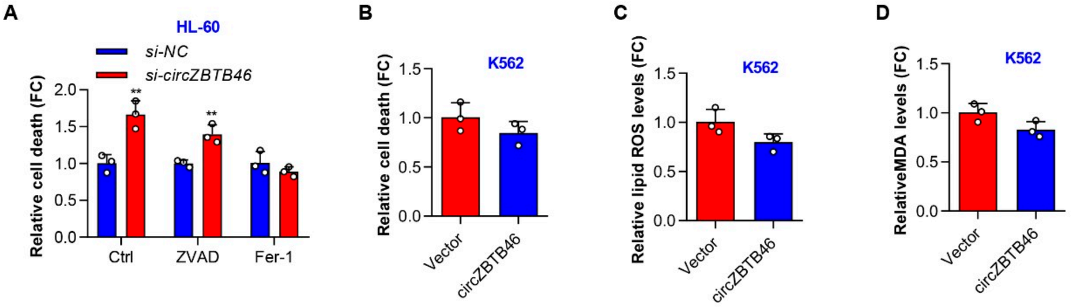

Figure S4. The effect of *circZBTB46* knockdown or overexpression on ferroptosis of AML cells. (A) Relative cell death of control and *circZBTB46*-knockdown cells in the absence or presence of ZVAD or ferrostatin-1. **(B)** Relative cell death of K562 cells after the transfection of the control vector or *circZBTB4* overexpression vector. **(C)** Relative lipid ROS levels of K562 cells after the transfection of the control vector or *circZBTB4* overexpression vector. ROS, reactive oxygen species. **(D)** Relative MDA levels of K562 cells after the transfection of the control vector or *circZBTB4* overexpression vector. MAD, malondialdehyde. The data are shown as the mean ± SD. The *P* values were determined by a two-tailed unpaired Student's t test; Related to Figure 4.

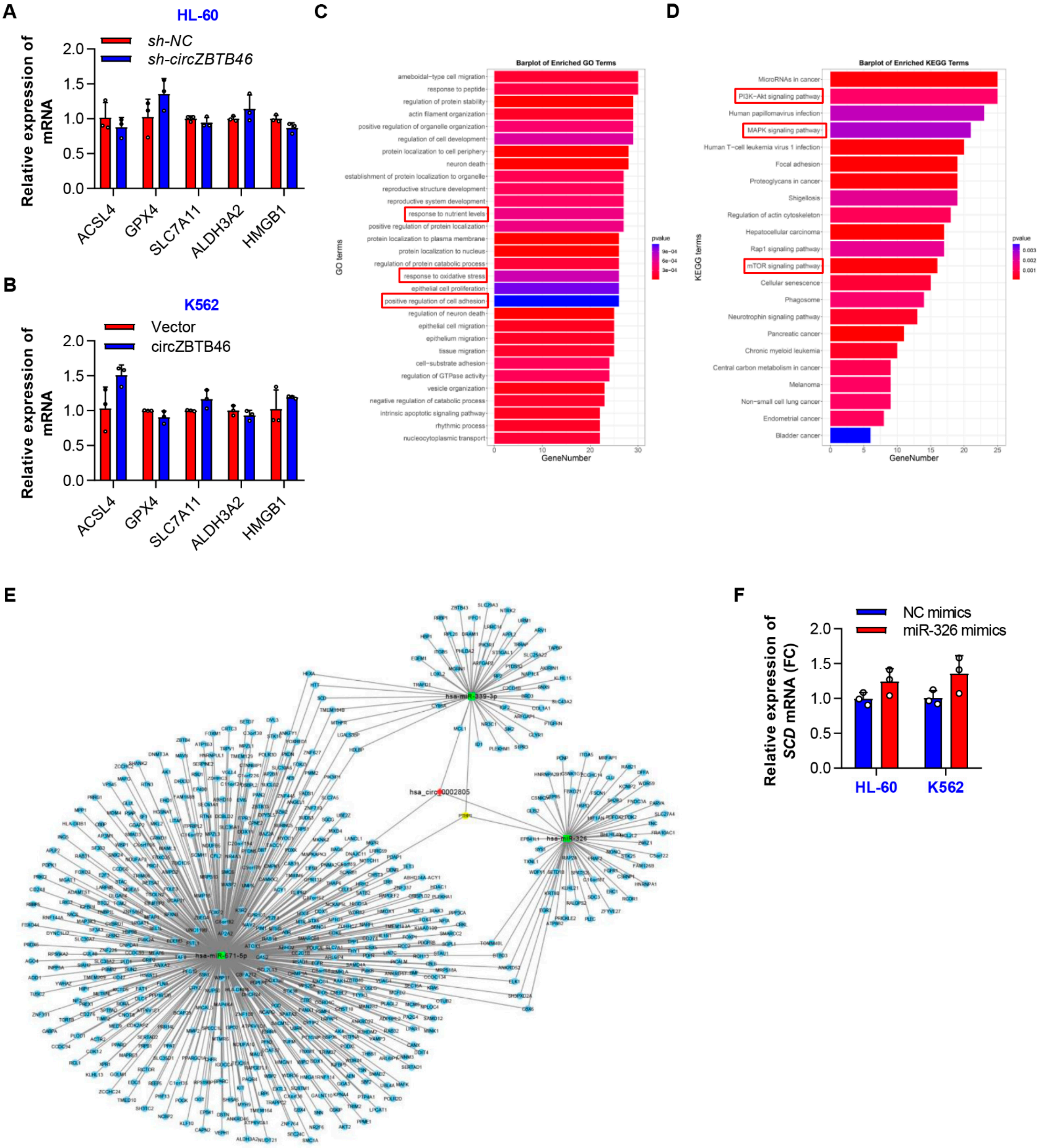

Figure S5. The expression of ferroptosis-related genes in AML cells with *circZBTB46* knockdown or overexpression. **(A)** qRT–PCR showing the expression of ferroptosis-related genes in HL-60 cells under control conditions (*sh-NC*) or upon *circZBTB46* knockdown (*sh-circZBTB46*). **(B)** qRT–PCR showing the expression of ferroptosis-related genes in K562 cells after the transfection of control vector or *circZBTB46* vector. **(C)** GO analysis annotates the biological process and clusters the modules of target genes. **(D)** KEGG analysis annotates the signaling pathway of target genes. **(E)** The crosstalk network of circRNA-miRNA-mRNA about *circZBTB46* (*hsa_circ_0002805*). **(F)** qRT–PCR showing the expression of *SCD* mRNA in AML cells after the transfection of NC mimics or miR-326 mimics. Related to Figure 5.

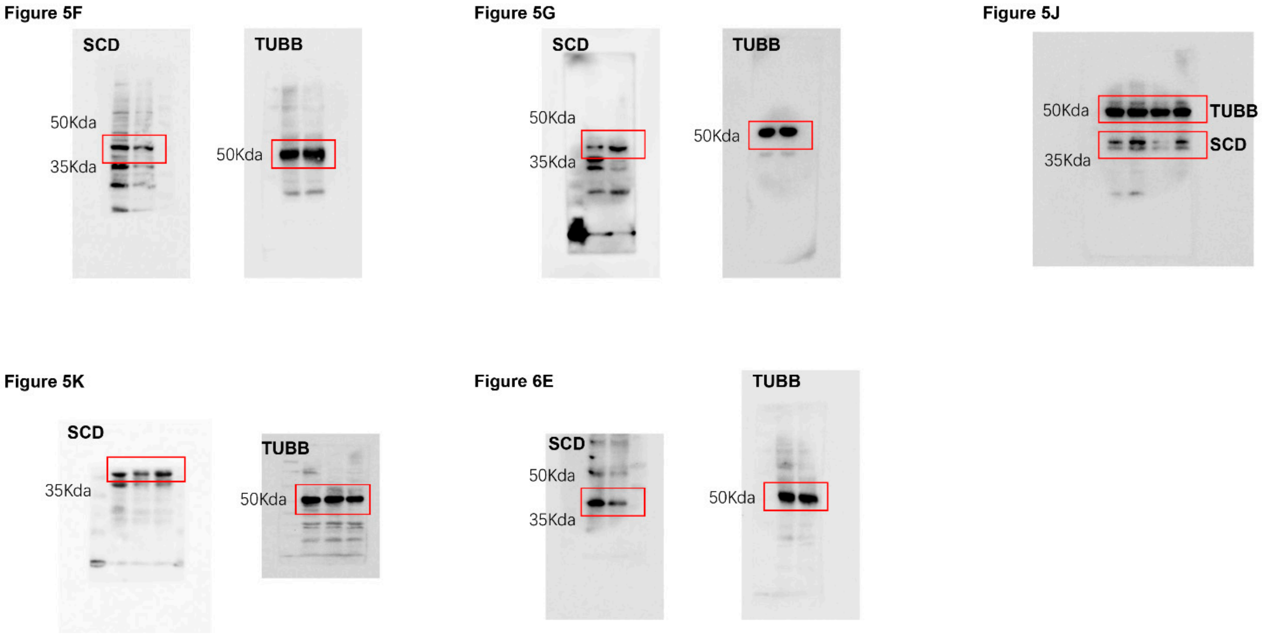

Figure S6. Uncropped Western blot images of Figure 5F-G, 5J-K, and 6E.

Table S1. The 13 and 68 significantly upregulated circRNAs screened from the two microarray datasets.

		**GSE94591**				
**CircRNA ID**	**logFC**	**AveExpr**	**t**	**P.Value**	**adj.P.Val**	**B**
hsa_circRNA_104336	2.085969	10.16515	4.645568	0.000716	0.00753	-0.46767
hsa_circRNA_001899	2.100628	7.765114	6.167206	7.14E-05	0.001649	1.883467
hsa_circRNA_104640	2.102201	14.02793	4.571876	0.000808	0.008012	-0.58982
hsa_circRNA_101287	2.142733	13.63376	3.627365	0.003996	0.023307	-2.2094
hsa_circRNA_103105	2.143185	7.316859	6.133585	7.48E-05	0.001672	1.835166
hsa_circRNA_103104	2.15949	8.362495	5.714889	0.000137	0.002524	1.219796
hsa_circRNA_102806	2.28082	8.525003	3.272134	0.007468	0.034692	-2.83573
hsa_circRNA_100033	2.477148	11.86404	4.481456	0.000937	0.00879	-0.74066
hsa_circRNA_101142	2.491151	12.22139	4.858889	0.000508	0.006086	-0.11812
hsa_circRNA_104181	2.79361	9.358879	4.274185	0.001321	0.011127	-1.09021
hsa_circRNA_102913	3.408693	11.15385	3.848818	0.002721	0.018009	-1.82216
hsa_circRNA_104700	3.680317	11.76521	6.884544	2.69E-05	0.000859	2.875286
hsa_circRNA_100199	4.779854	8.83435	9.801448	9.27E-07	0.000161	6.229259

		**GSE163386**				
**CircRNA ID**	**logFC**	**AveExpr**	**t**	**P.Value**	**adj.P.Val**	**B**
hsa_circRNA_101463	2.047413	7.794814	6.053828	0.000221	0.005869	0.698072
hsa_circRNA_101113	2.057848	7.321678	2.825025	0.020614	0.06961	-4.05447
hsa_circRNA_104821	2.072789	8.38212	3.885537	0.003973	0.026379	-2.35603
hsa_circRNA_103433	2.078558	7.383744	2.451518	0.037625	0.099281	-4.65741
hsa_circRNA_102315	2.108835	7.769759	3.71446	0.005136	0.030787	-2.624
hsa_circRNA_001059	2.123086	10.93351	3.747145	0.004888	0.029769	-2.57255
hsa_circRNA_102901	2.124669	7.389081	4.506969	0.001619	0.016692	-1.41299
hsa_circRNA_104948	2.128567	7.935827	4.506407	0.001621	0.016692	-1.41382
hsa_circRNA_104833	2.142165	9.151013	5.665561	0.000353	0.007672	0.200433
hsa_circRNA_103312	2.147661	7.803034	3.587518	0.006228	0.034526	-2.82483
hsa_circRNA_100010	2.174446	8.199833	3.439418	0.007821	0.039549	-3.06102
hsa_circRNA_104792	2.194531	8.039298	6.167558	0.000193	0.005516	0.839878
hsa_circRNA_101172	2.204741	7.538335	4.499624	0.001636	0.016698	-1.42383
hsa_circRNA_001622	2.212779	7.618225	3.11559	0.012977	0.053503	-3.58293
hsa_circRNA_101488	2.214877	7.421224	8.598035	1.57E-05	0.001438	3.487348
hsa_circRNA_103104	2.235255	7.743211	2.936719	0.017241	0.062843	-3.87312
hsa_circRNA_103176	2.236382	8.320926	3.482617	0.007316	0.037941	-2.99193
hsa_circRNA_100003	2.238126	7.664472	4.078659	0.002988	0.02265	-2.05762
hsa_circRNA_101314	2.239374	8.064184	5.683499	0.000345	0.007614	0.223891
hsa_circRNA_100583	2.2626	7.59768	3.790368	0.004581	0.028726	-2.5047
hsa_circRNA_103695	2.294603	8.463686	3.963396	0.00354	0.024392	-2.23518

hsa_circRNA_103514	2.29468	8.261188	4.215355	0.002451	0.020182	-1.84922
hsa_circRNA_101555	2.311228	12.09465	4.070237	0.003025	0.022749	-2.07054
hsa_circRNA_100245	2.355684	7.765309	16.2735	8.47E-08	3.66E-05	8.736009
hsa_circRNA_002172	2.359638	13.22357	2.364296	0.043315	0.108886	-4.79646
hsa_circRNA_101033	2.370043	7.730358	3.608641	0.006031	0.033864	-2.7913
hsa_circRNA_104479	2.37709	7.948591	3.960122	0.003557	0.024462	-2.24025
hsa_circRNA_100016	2.396914	8.884754	3.447525	0.007723	0.039228	-3.04804
hsa_circRNA_103435	2.415665	8.445025	3.641702	0.005735	0.03306	-2.73891
hsa_circRNA_103619	2.419435	9.628518	2.971309	0.016316	0.060607	-3.81696
hsa_circRNA_103870	2.449179	8.161759	4.984276	0.000844	0.01222	-0.72479
hsa_circRNA_100015	2.465624	8.663063	3.547974	0.006617	0.035679	-2.88771
hsa_circRNA_103045	2.469936	8.051282	4.229251	0.002402	0.019877	-1.82817
hsa_circRNA_000274	2.470078	11.47182	3.10225	0.013254	0.054	-3.60455
hsa_circRNA_001846	2.480501	10.54641	2.525534	0.033386	0.09234	-4.53872
hsa_circRNA_100012	2.510892	9.078743	3.781007	0.004646	0.028797	-2.51938
hsa_circRNA_002178	2.519702	11.99667	2.856198	0.01961	0.067492	-4.00387
hsa_circRNA_100517	2.537771	7.858853	7.087848	6.93E-05	0.003535	1.924587
hsa_circRNA_103407	2.550007	8.119292	4.29058	0.0022	0.01916	-1.73559
hsa_circRNA_000585	2.572173	11.53672	2.785044	0.021979	0.071588	-4.11934
hsa_circRNA_101835	2.605704	9.300626	3.071004	0.013926	0.055496	-3.65519
hsa_circRNA_103108	2.627798	8.324777	3.531554	0.006786	0.036166	-2.91386
hsa_circRNA_105028	2.737336	7.793983	3.553986	0.006556	0.035565	-2.87814
hsa_circRNA_000166	2.761218	12.56512	2.529461	0.033175	0.091921	-4.53241
hsa_circRNA_102712	2.815735	8.131966	5.28043	0.000573	0.010011	-0.31434
hsa_circRNA_103871	2.832531	8.217798	4.321112	0.002106	0.018676	-1.68968
hsa_circRNA_104645	2.83647	9.689801	3.558174	0.006514	0.035541	-2.87147
hsa_circRNA_002144	2.837792	12.38236	2.657171	0.026999	0.081536	-4.32647
hsa_circRNA_100631	2.838253	8.205656	3.732652	0.004996	0.030238	-2.59535
hsa_circRNA_100414	2.894534	7.857998	3.644471	0.005711	0.032976	-2.73453
hsa_circRNA_101836	2.982769	9.317172	3.424075	0.008008	0.040171	-3.08559
hsa_circRNA_104634	2.993671	8.127091	10.33071	3.64E-06	0.000629	5.006874
hsa_circRNA_101903	2.998643	9.407808	3.692056	0.005313	0.031467	-2.65932
hsa_circRNA_104671	3.002265	9.637184	4.15245	0.002684	0.021293	-1.94482
hsa_circRNA_100632	3.038505	8.219504	3.75151	0.004856	0.029679	-2.56569
hsa_circRNA_100679	3.069502	10.15601	5.144165	0.000684	0.010807	-0.50161
hsa_circRNA_103499	3.08827	8.445489	6.500391	0.000132	0.004555	1.244849
hsa_circRNA_104435	3.089381	8.961581	3.693905	0.005298	0.031433	-2.65641
hsa_circRNA_100097	3.117204	8.479481	4.893515	0.000953	0.012835	-0.85312
hsa_circRNA_103017	3.162877	9.652526	3.526786	0.006836	0.036356	-2.92145
hsa_circRNA_002111	3.165186	8.779666	6.037511	0.000225	0.005869	0.67758
hsa_circRNA_001379	3.189428	10.70057	4.873431	0.000979	0.012925	-0.88168
hsa_circRNA_104766	3.350103	10.02848	4.155147	0.002673	0.021259	-1.94071
hsa_circRNA_100199	3.378275	7.777817	3.544367	0.006654	0.035793	-2.89345
hsa_circRNA_001678	3.495568	10.57042	3.9136	0.003811	0.025687	-2.31239
hsa_circRNA_000167	3.502511	11.04242	3.356581	0.008893	0.043385	-3.19391
hsa_circRNA_104056	3.54755	8.575609	5.707628	0.000335	0.00757	0.255373
hsa_circRNA_104547	4.237441	9.770693	5.242095	0.000602	0.010322	-0.36675

Table S2. The target genes of the 3 miRNAs predicted by miRTarBase, ENCORI, and TargetScan databases.

miRNAName	geneName
hsa-miR-326	KLHL21
hsa-miR-326	DFFA
hsa-miR-326	SDC3
hsa-miR-326	H6PD
hsa-miR-326	CEP85
hsa-miR-326	ATP8B2
hsa-miR-326	TOMM40L
hsa-miR-326	RALGPS2
hsa-miR-326	FRA10AC1
hsa-miR-326	KCNIP2
hsa-miR-326	SH3PXD2A
hsa-miR-326	ZMIZ1
hsa-miR-326	ZFYVE27
hsa-miR-326	HIF1AN

hsa-miR-326	PARVA
hsa-miR-326	KRT80
hsa-miR-326	ITGA5
hsa-miR-326	ANKRD52
hsa-miR-326	FBXO21
hsa-miR-326	ERC1
hsa-miR-326	HNRNPA1
hsa-miR-326	RAB21
hsa-miR-326	SH2B3
hsa-miR-326	SETD1B
hsa-miR-326	FNDC3A
hsa-miR-326	RAP2A
hsa-miR-326	BCL2L2
hsa-miR-326	RCOR1
hsa-miR-326	CSNK1G1
hsa-miR-326	C16orf87
hsa-miR-326	WDR59
hsa-miR-326	ZCCHC14
hsa-miR-326	GLIS2
hsa-miR-326	PDK2
hsa-miR-326	TXNL1
hsa-miR-326	PTBP1
hsa-miR-326	FAM126B
hsa-miR-326	WDFY1
hsa-miR-326	STK25
hsa-miR-326	SPATS2L
hsa-miR-326	CSNK2A1
hsa-miR-326	BTBD3
hsa-miR-326	EPB41L1
hsa-miR-326	SYS1
hsa-miR-326	RBFOX2
hsa-miR-326	CBX6
hsa-miR-326	CSRNP1
hsa-miR-326	PRICKLE2
hsa-miR-326	BHLHE40
hsa-miR-326	PCNP
hsa-miR-326	MRFAP1
hsa-miR-326	C5orf22
hsa-miR-326	HNRNPA2B1
hsa-miR-326	FSCN1
hsa-miR-326	EGR3
hsa-miR-326	CLU
hsa-miR-326	FGFR1
hsa-miR-326	PLEC
hsa-miR-326	TNC
hsa-miR-326	SLC27A4
hsa-miR-326	ELK1
hsa-miR-326	PRAF2
hsa-miR-326	NONO
hsa-miR-339-3p	MTHFR
hsa-miR-339-3p	MCL1
hsa-miR-339-3p	AKIRIN1
hsa-miR-339-3p	PTGFRN
hsa-miR-339-3p	ARV1
hsa-miR-339-3p	SLC29A3
hsa-miR-339-3p	SCD
hsa-miR-339-3p	SLC25A22
hsa-miR-339-3p	IGF2

hsa-miR-339-3p	PHLDA2
hsa-miR-339-3p	NAP1L4
hsa-miR-339-3p	ARFGAP2
hsa-miR-339-3p	PTDSS2
hsa-miR-339-3p	SIK2
hsa-miR-339-3p	IFFO1
hsa-miR-339-3p	APPL2
hsa-miR-339-3p	DRAM1
hsa-miR-339-3p	TRAFD1
hsa-miR-339-3p	C2CD4B
hsa-miR-339-3p	HEXA
hsa-miR-339-3p	GLYR1
hsa-miR-339-3p	MGRN1
hsa-miR-339-3p	SLC43A2
hsa-miR-339-3p	PLEKHM1
hsa-miR-339-3p	COL1A1
hsa-miR-339-3p	LGALS3BP
hsa-miR-339-3p	CYB5A
hsa-miR-339-3p	PTBP1
hsa-miR-339-3p	RPL28
hsa-miR-339-3p	HDLBP
hsa-miR-339-3p	RRBP1
hsa-miR-339-3p	ID1
hsa-miR-339-3p	ARFGAP1
hsa-miR-339-3p	TMEM184B
hsa-miR-339-3p	ITGB5
hsa-miR-339-3p	EDEM1
hsa-miR-339-3p	HTT
hsa-miR-339-3p	NR3C1
hsa-miR-339-3p	PIK3R1
hsa-miR-339-3p	TAPBP
hsa-miR-339-3p	SNX9
hsa-miR-339-3p	TRRAP
hsa-miR-339-3p	HBP1
hsa-miR-339-3p	LOXL2
hsa-miR-339-3p	ST3GAL1
hsa-miR-339-3p	LRRC14
hsa-miR-339-3p	BRD3
hsa-miR-339-3p	NTRK2
hsa-miR-339-3p	S1PR3
hsa-miR-339-3p	ZBTB43
hsa-miR-339-3p	URM1
hsa-miR-339-3p	KLHL15
hsa-miR-339-3p	RP2
hsa-miR-671-5p	DNAJC11
hsa-miR-671-5p	CTNNBIP1
hsa-miR-671-5p	MTHFR
hsa-miR-671-5p	RCC2
hsa-miR-671-5p	UBR4
hsa-miR-671-5p	WASF2
hsa-miR-671-5p	PSMB2
hsa-miR-671-5p	MEAF6
hsa-miR-671-5p	SF3A3
hsa-miR-671-5p	SCMH1
hsa-miR-671-5p	FOXJ3
hsa-miR-671-5p	CC2D1B
hsa-miR-671-5p	DHCR24
hsa-miR-671-5p	SLC35D1

hsa-miR-671-5p	CTBS
hsa-miR-671-5p	EVI5
hsa-miR-671-5p	DBT
hsa-miR-671-5p	GDAP2
hsa-miR-671-5p	PI4KB
hsa-miR-671-5p	SMG5
hsa-miR-671-5p	HDGF
hsa-miR-671-5p	STX6
hsa-miR-671-5p	GLUL
hsa-miR-671-5p	EDEM3
hsa-miR-671-5p	RBBP5
hsa-miR-671-5p	LPGAT1
hsa-miR-671-5p	C1orf35
hsa-miR-671-5p	PHF13
hsa-miR-671-5p	VAMP3
hsa-miR-671-5p	PGD
hsa-miR-671-5p	FBXO44
hsa-miR-671-5p	PLOD1
hsa-miR-671-5p	ZBTB40
hsa-miR-671-5p	SESN2
hsa-miR-671-5p	EPB41
hsa-miR-671-5p	HDAC1
hsa-miR-671-5p	AGO4
hsa-miR-671-5p	ADPRHL2
hsa-miR-671-5p	RIMKLA
hsa-miR-671-5p	CMPK1
hsa-miR-671-5p	NFIA
hsa-miR-671-5p	AK4
hsa-miR-671-5p	FNBP1L
hsa-miR-671-5p	SLC30A7
hsa-miR-671-5p	AMPD2
hsa-miR-671-5p	LRIG2
hsa-miR-671-5p	VPS45
hsa-miR-671-5p	TOMM40L
hsa-miR-671-5p	C1orf226
hsa-miR-671-5p	POGK
hsa-miR-671-5p	MPZL1
hsa-miR-671-5p	PRDX6
hsa-miR-671-5p	XPR1
hsa-miR-671-5p	PPP1R12B
hsa-miR-671-5p	TMEM183A
hsa-miR-671-5p	BTG2
hsa-miR-671-5p	MDM4
hsa-miR-671-5p	CAPN2
hsa-miR-671-5p	MTR
hsa-miR-671-5p	LARP4B
hsa-miR-671-5p	RSU1
hsa-miR-671-5p	CCDC6
hsa-miR-671-5p	PSAP
hsa-miR-671-5p	AP3M1
hsa-miR-671-5p	ZCCHC24
hsa-miR-671-5p	MGEA5
hsa-miR-671-5p	SH3PXD2A
hsa-miR-671-5p	RAB18
hsa-miR-671-5p	VPS26A
hsa-miR-671-5p	EIF4EBP2
hsa-miR-671-5p	SGPL1
hsa-miR-671-5p	CHST3

hsa-miR-671-5p	DDIT4
hsa-miR-671-5p	SEC24C
hsa-miR-671-5p	PI4K2A
hsa-miR-671-5p	SCD
hsa-miR-671-5p	SFXN3
hsa-miR-671-5p	PLEKHA1
hsa-miR-671-5p	INPP5A
hsa-miR-671-5p	TPP1
hsa-miR-671-5p	TRIM66
hsa-miR-671-5p	SBF2
hsa-miR-671-5p	LIN7C
hsa-miR-671-5p	CELF1
hsa-miR-671-5p	OSBP
hsa-miR-671-5p	FADS1
hsa-miR-671-5p	SF1
hsa-miR-671-5p	EHD1
hsa-miR-671-5p	CD248
hsa-miR-671-5p	SPTBN2
hsa-miR-671-5p	CDK2AP2
hsa-miR-671-5p	C11orf24
hsa-miR-671-5p	SHANK2
hsa-miR-671-5p	PICALM
hsa-miR-671-5p	MPZL3
hsa-miR-671-5p	HYOU1
hsa-miR-671-5p	THY1
hsa-miR-671-5p	AP2A2
hsa-miR-671-5p	NAV2
hsa-miR-671-5p	CRY2
hsa-miR-671-5p	RTN3
hsa-miR-671-5p	GPR137
hsa-miR-671-5p	ESRRA
hsa-miR-671-5p	PPME1
hsa-miR-671-5p	PANX1
hsa-miR-671-5p	FOXRED1
hsa-miR-671-5p	APLP2
hsa-miR-671-5p	FOXM1
hsa-miR-671-5p	M6PR
hsa-miR-671-5p	LRP6
hsa-miR-671-5p	WBP11
hsa-miR-671-5p	KRAS
hsa-miR-671-5p	SLC38A2
hsa-miR-671-5p	NCKAP5L
hsa-miR-671-5p	CBX5
hsa-miR-671-5p	SMARCC2
hsa-miR-671-5p	ANKRD52
hsa-miR-671-5p	PAN2
hsa-miR-671-5p	BAZ2A
hsa-miR-671-5p	R3HDM2
hsa-miR-671-5p	CORO1C
hsa-miR-671-5p	KSR2
hsa-miR-671-5p	CAMKK2
hsa-miR-671-5p	SBNO1
hsa-miR-671-5p	SCARB1
hsa-miR-671-5p	CHFR
hsa-miR-671-5p	CREBL2
hsa-miR-671-5p	DDX11
hsa-miR-671-5p	TMBIM6
hsa-miR-671-5p	PFDN5

hsa-miR-671-5p	PA2G4
hsa-miR-671-5p	IRAK3
hsa-miR-671-5p	OAS2
hsa-miR-671-5p	SUDS3
hsa-miR-671-5p	HSPB8
hsa-miR-671-5p	UNC119B
hsa-miR-671-5p	DENR
hsa-miR-671-5p	ARL6IP4
hsa-miR-671-5p	EP400
hsa-miR-671-5p	SACS
hsa-miR-671-5p	MTMR6
hsa-miR-671-5p	SLC7A1
hsa-miR-671-5p	SGCG
hsa-miR-671-5p	IPO5
hsa-miR-671-5p	CFL2
hsa-miR-671-5p	TMED10
hsa-miR-671-5p	ANGEL1
hsa-miR-671-5p	SEL1L
hsa-miR-671-5p	BAG5
hsa-miR-671-5p	TOX4
hsa-miR-671-5p	SAMD4A
hsa-miR-671-5p	OTUB2
hsa-miR-671-5p	GSKIP
hsa-miR-671-5p	CRIP2
hsa-miR-671-5p	BMF
hsa-miR-671-5p	MFAP1
hsa-miR-671-5p	ANXA2
hsa-miR-671-5p	RORA
hsa-miR-671-5p	IGDCC4
hsa-miR-671-5p	HEXA
hsa-miR-671-5p	EDC3
hsa-miR-671-5p	PRC1
hsa-miR-671-5p	THBS1
hsa-miR-671-5p	SMAD3
hsa-miR-671-5p	CD276
hsa-miR-671-5p	AEN
hsa-miR-671-5p	ABHD2
hsa-miR-671-5p	ZNF710
hsa-miR-671-5p	CRTC3
hsa-miR-671-5p	NARFL
hsa-miR-671-5p	LITAF
hsa-miR-671-5p	ARL6IP1
hsa-miR-671-5p	TUFM
hsa-miR-671-5p	ZNF764
hsa-miR-671-5p	NUDT21
hsa-miR-671-5p	DYNC1LI2
hsa-miR-671-5p	AP1G1
hsa-miR-671-5p	ZDHHC7
hsa-miR-671-5p	SLC7A5
hsa-miR-671-5p	CHMP1A
hsa-miR-671-5p	PDPK1
hsa-miR-671-5p	KCTD5
hsa-miR-671-5p	PAQR4
hsa-miR-671-5p	PMM2
hsa-miR-671-5p	C16orf72
hsa-miR-671-5p	SNN
hsa-miR-671-5p	MKL2
hsa-miR-671-5p	ITPRIPL2

hsa-miR-671-5p	MMP2
hsa-miR-671-5p	CPNE2
hsa-miR-671-5p	PDPR
hsa-miR-671-5p	SF3B3
hsa-miR-671-5p	CRISPLD2
hsa-miR-671-5p	ABR
hsa-miR-671-5p	PITPNA
hsa-miR-671-5p	TRPV1
hsa-miR-671-5p	ANKFY1
hsa-miR-671-5p	DHX33
hsa-miR-671-5p	ZBTB4
hsa-miR-671-5p	KIAA0100
hsa-miR-671-5p	LRRC59
hsa-miR-671-5p	VEZF1
hsa-miR-671-5p	DDX5
hsa-miR-671-5p	GGA3
hsa-miR-671-5p	WBP2
hsa-miR-671-5p	USP36
hsa-miR-671-5p	TIMP2
hsa-miR-671-5p	LGALS3BP
hsa-miR-671-5p	CBX4
hsa-miR-671-5p	NPLOC4
hsa-miR-671-5p	MAFG
hsa-miR-671-5p	C17orf62
hsa-miR-671-5p	MINK1
hsa-miR-671-5p	MED9
hsa-miR-671-5p	ALDH3A2
hsa-miR-671-5p	AP2B1
hsa-miR-671-5p	CDK12
hsa-miR-671-5p	RAPGEFL1
hsa-miR-671-5p	IGFBP4
hsa-miR-671-5p	ATP6V0A1
hsa-miR-671-5p	UBE2Z
hsa-miR-671-5p	RSAD1
hsa-miR-671-5p	MAP3K3
hsa-miR-671-5p	METRNL
hsa-miR-671-5p	SMAD2
hsa-miR-671-5p	RAB31
hsa-miR-671-5p	ZCCHC2
hsa-miR-671-5p	POLR2E
hsa-miR-671-5p	MKNK2
hsa-miR-671-5p	AES
hsa-miR-671-5p	NR2F6
hsa-miR-671-5p	ZNF100
hsa-miR-671-5p	ANKRD27
hsa-miR-671-5p	ZNF585B
hsa-miR-671-5p	AKT2
hsa-miR-671-5p	SERTAD1
hsa-miR-671-5p	RDH13
hsa-miR-671-5p	PTBP1
hsa-miR-671-5p	CCDC94
hsa-miR-671-5p	ZNF627
hsa-miR-671-5p	NACC1
hsa-miR-671-5p	PGPEP1
hsa-miR-671-5p	MAU2
hsa-miR-671-5p	HNRNPUL1
hsa-miR-671-5p	ZNF225
hsa-miR-671-5p	GRWD1

hsa-miR-671-5p	PXDN
hsa-miR-671-5p	DNMT3A
hsa-miR-671-5p	MCFD2
hsa-miR-671-5p	RTN4
hsa-miR-671-5p	SERTAD2
hsa-miR-671-5p	AAK1
hsa-miR-671-5p	TEX261
hsa-miR-671-5p	TGOLN2
hsa-miR-671-5p	RETSAT
hsa-miR-671-5p	CHST10
hsa-miR-671-5p	CCDC93
hsa-miR-671-5p	POLR2D
hsa-miR-671-5p	HS6ST1
hsa-miR-671-5p	FAM168B
hsa-miR-671-5p	MCM6
hsa-miR-671-5p	SATB2
hsa-miR-671-5p	LANCL1
hsa-miR-671-5p	IGFBP5
hsa-miR-671-5p	SERPINE2
hsa-miR-671-5p	NDUFA10
hsa-miR-671-5p	HDLBP
hsa-miR-671-5p	RNF144A
hsa-miR-671-5p	DDX1
hsa-miR-671-5p	DPYSL5
hsa-miR-671-5p	MAPRE3
hsa-miR-671-5p	SLC30A6
hsa-miR-671-5p	ACTR2
hsa-miR-671-5p	TET3
hsa-miR-671-5p	CNNM3
hsa-miR-671-5p	EIF5B
hsa-miR-671-5p	MAP4K4
hsa-miR-671-5p	IL1R1
hsa-miR-671-5p	TSN
hsa-miR-671-5p	GPD2
hsa-miR-671-5p	DCAF17
hsa-miR-671-5p	CYBRD1
hsa-miR-671-5p	STK16
hsa-miR-671-5p	CAB39
hsa-miR-671-5p	ING5
hsa-miR-671-5p	C20orf194
hsa-miR-671-5p	ZNF337
hsa-miR-671-5p	PLAGL2
hsa-miR-671-5p	TGM2
hsa-miR-671-5p	PREX1
hsa-miR-671-5p	STAU1
hsa-miR-671-5p	SPATA2
hsa-miR-671-5p	PSMF1
hsa-miR-671-5p	MAVS
hsa-miR-671-5p	BTBD3
hsa-miR-671-5p	DSTN
hsa-miR-671-5p	CBFA2T2
hsa-miR-671-5p	DLGAP4
hsa-miR-671-5p	RNF114
hsa-miR-671-5p	OSBPL2
hsa-miR-671-5p	ADAMTS1
hsa-miR-671-5p	HMGN1
hsa-miR-671-5p	ICOSLG
hsa-miR-671-5p	PTTG1IP

hsa-miR-671-5p	GABPA
hsa-miR-671-5p	PDXK
hsa-miR-671-5p	ATP6V1E1
hsa-miR-671-5p	MICAL3
hsa-miR-671-5p	PIK3IP1
hsa-miR-671-5p	PRR14L
hsa-miR-671-5p	MYH9
hsa-miR-671-5p	TMEM184B
hsa-miR-671-5p	SUN2
hsa-miR-671-5p	CBX6
hsa-miR-671-5p	RPS19BP1
hsa-miR-671-5p	BCL2L13
hsa-miR-671-5p	CRKL
hsa-miR-671-5p	SMARCB1
hsa-miR-671-5p	SPECC1L
hsa-miR-671-5p	NF2
hsa-miR-671-5p	HMOX1
hsa-miR-671-5p	CCDC134
hsa-miR-671-5p	NUP50
hsa-miR-671-5p	ZBED4
hsa-miR-671-5p	VGLL4
hsa-miR-671-5p	TBC1D5
hsa-miR-671-5p	HIGD1A
hsa-miR-671-5p	ZNF445
hsa-miR-671-5p	ZDHHC3
hsa-miR-671-5p	SHISA5
hsa-miR-671-5p	TUSC2
hsa-miR-671-5p	WDR82
hsa-miR-671-5p	SUCLG2
hsa-miR-671-5p	FOXP1
hsa-miR-671-5p	DCBLD2
hsa-miR-671-5p	CBLB
hsa-miR-671-5p	CD47
hsa-miR-671-5p	FSTL1
hsa-miR-671-5p	PFN2
hsa-miR-671-5p	SIAH2
hsa-miR-671-5p	VEPH1
hsa-miR-671-5p	KPNA4
hsa-miR-671-5p	PLD1
hsa-miR-671-5p	NCBP2
hsa-miR-671-5p	NR2C2
hsa-miR-671-5p	STAC
hsa-miR-671-5p	SACM1L
hsa-miR-671-5p	NDUFAF3
hsa-miR-671-5p	MAPKAPK3
hsa-miR-671-5p	ABHD14A-ACY1
hsa-miR-671-5p	ACY1
hsa-miR-671-5p	C3orf38
hsa-miR-671-5p	ABHD10
hsa-miR-671-5p	UMPS
hsa-miR-671-5p	SEC61A1
hsa-miR-671-5p	ATP1B3
hsa-miR-671-5p	PRKCI
hsa-miR-671-5p	NDUFB5
hsa-miR-671-5p	DVL3
hsa-miR-671-5p	TMEM129
hsa-miR-671-5p	MXD4
hsa-miR-671-5p	LRPAP1

hsa-miR-671-5p	PPARGC1A
hsa-miR-671-5p	PPAT
hsa-miR-671-5p	ANKRD17
hsa-miR-671-5p	PPP3CA
hsa-miR-671-5p	SETD7
hsa-miR-671-5p	MAML3
hsa-miR-671-5p	FBXW7
hsa-miR-671-5p	FAT1
hsa-miR-671-5p	ADD1
hsa-miR-671-5p	HTT
hsa-miR-671-5p	MAN2B2
hsa-miR-671-5p	NCAPG
hsa-miR-671-5p	KIT
hsa-miR-671-5p	THAP6
hsa-miR-671-5p	TRIM2
hsa-miR-671-5p	RAPGEF2
hsa-miR-671-5p	LPCAT1
hsa-miR-671-5p	RICTOR
hsa-miR-671-5p	WDR41
hsa-miR-671-5p	SERINC5
hsa-miR-671-5p	REEP5
hsa-miR-671-5p	VDAC1
hsa-miR-671-5p	GNPDA1
hsa-miR-671-5p	SH3TC2
hsa-miR-671-5p	PDGFRB
hsa-miR-671-5p	SPARC
hsa-miR-671-5p	ATOX1
hsa-miR-671-5p	MGAT1
hsa-miR-671-5p	MTRR
hsa-miR-671-5p	WDR36
hsa-miR-671-5p	SNX24
hsa-miR-671-5p	SLC36A1
hsa-miR-671-5p	GALNT10
hsa-miR-671-5p	CNOT8
hsa-miR-671-5p	CYFIP2
hsa-miR-671-5p	HRH2
hsa-miR-671-5p	CANX
hsa-miR-671-5p	SQSTM1
hsa-miR-671-5p	SSR1
hsa-miR-671-5p	TRIM27
hsa-miR-671-5p	DDX39B
hsa-miR-671-5p	HLA-DRB5
hsa-miR-671-5p	HLA-DRB1
hsa-miR-671-5p	C6orf106
hsa-miR-671-5p	STK38
hsa-miR-671-5p	MRPS10
hsa-miR-671-5p	MRPS18A
hsa-miR-671-5p	RPS6KA2
hsa-miR-671-5p	E2F3
hsa-miR-671-5p	ZNF391
hsa-miR-671-5p	ITPR3
hsa-miR-671-5p	HMGA1
hsa-miR-671-5p	PPARD
hsa-miR-671-5p	C6orf89
hsa-miR-671-5p	PIM1
hsa-miR-671-5p	TAF8
hsa-miR-671-5p	PTP4A1
hsa-miR-671-5p	FOXO3

hsa-miR-671-5p	RAB32
hsa-miR-671-5p	HIP1
hsa-miR-671-5p	CDK6
hsa-miR-671-5p	PDAP1
hsa-miR-671-5p	TMEM209
hsa-miR-671-5p	PODXL
hsa-miR-671-5p	MAFK
hsa-miR-671-5p	TTYH3
hsa-miR-671-5p	WIPI2
hsa-miR-671-5p	BZW2
hsa-miR-671-5p	EGFR
hsa-miR-671-5p	PEG10
hsa-miR-671-5p	BCAP29
hsa-miR-671-5p	FOXP2
hsa-miR-671-5p	DLC1
hsa-miR-671-5p	PNMA2
hsa-miR-671-5p	MMP16
hsa-miR-671-5p	ANKRD46
hsa-miR-671-5p	YWHAZ
hsa-miR-671-5p	KLF10
hsa-miR-671-5p	TRPS1
hsa-miR-671-5p	SAMD12
hsa-miR-671-5p	C8orf82
hsa-miR-671-5p	FBXO25
hsa-miR-671-5p	ERI1
hsa-miR-671-5p	POLR3D
hsa-miR-671-5p	C8orf58
hsa-miR-671-5p	EXTL3
hsa-miR-671-5p	TACC1
hsa-miR-671-5p	HGSNAT
hsa-miR-671-5p	ATP6V1C1
hsa-miR-671-5p	WISP1
hsa-miR-671-5p	GOLM1
hsa-miR-671-5p	LPAR1
hsa-miR-671-5p	POLE3
hsa-miR-671-5p	AK1
hsa-miR-671-5p	C9orf78
hsa-miR-671-5p	SEC16A
hsa-miR-671-5p	NOTCH1
hsa-miR-671-5p	RCL1
hsa-miR-671-5p	CD274
hsa-miR-671-5p	GLIPR2
hsa-miR-671-5p	NR4A3
hsa-miR-671-5p	TOR1B
hsa-miR-671-5p	GPR107
hsa-miR-671-5p	TRAPPC2
hsa-miR-671-5p	CXorf38
hsa-miR-671-5p	ELK1
hsa-miR-671-5p	SMC1A
hsa-miR-671-5p	KLHL13
hsa-miR-671-5p	CUL4B
hsa-miR-671-5p	BCAP31
hsa-miR-671-5p	FLNA
hsa-miR-671-5p	UBL4A
hsa-miR-671-5p	MPP1
hsa-miR-671-5p	PRRG1
hsa-miR-671-5p	OGT
hsa-miR-671-5p	PRPS1

hsa-miR-671-5p	TMEM164
hsa-miR-671-5p	ZBTB33
hsa-miR-339-3p	hsa_circ_0002805
hsa-miR-326	hsa_circ_0002805
hsa-miR-671-5p	hsa_circ_0002805
